# Rac1 GTPase Regulates the SCF^βTrCP^-Mediated Degradation of Claspin and the Cellular Response of Pancreatic Cancer Cells to Gamma Rays

**DOI:** 10.3390/cancers18121908

**Published:** 2026-06-11

**Authors:** Neha Chaudhary, Tabbatha N. Somers, Surinder K. Batra, Ying Yan, Michel M. Ouellette

**Affiliations:** 1 Department Internal Medicine, University of Nebraska Medical Center, Omaha, NE 68198, USA; chaudhary19neha@gmail.com (N.C.); tsomers@unmc.edu (T.N.S.); 2 Department of Biochemistry and Molecular Biology, University of Nebraska Medical Center, Omaha, NE 68198, USA; sbatra@unmc.edu; 3 Department of Radiation Oncology, University of Nebraska Medical Center, Omaha, NE 68198, USA; yyan@unmc.edu; 4 Department of Genetics, Cell Biology and Anatomy, University of Nebraska Medical Center, Omaha, NE 68198, USA

**Keywords:** Claspin, Rac1, pancreatic cancer, ubiquitin ligases, βTrCP, ATR, Chk1, GTPase

## Abstract

Background/Objectives: Pancreatic ductal adenocarcinomas (PDACs) are lethal tumors exhibiting resistance to most cancer therapeutics, particularly DNA-damaging agents. The *KRAS* oncogene drives PDACs, and many of these tumors are addicted to it and its downstream effectors. One such effector is Rac1, a small GTPase involved in actin cytoskeleton remodeling and regulation of the DNA damage response. We previously showed that Rac1 inhibition blocks activation of ATM/Chk2 and ATR/Chk1 pathways in response to gamma rays, sensitizing PDAC cells to radiation. Methods: Western blot analyses were used to assess the impacts of Rac1 inhibition on the components of the ATR/Chk1 cascade. Results: Here, we show that Rac1 inhibition disrupts ATR/Chk1 signaling by promoting degradation of Claspin, a key component of the fork protection complex needed for the Ser345-phosphorylation of Chk1 by ATR. In PDACs and normal pancreatic ductal cells, Rac1 inhibition (via inhibitors or siRNA) decreased Claspin protein levels without affecting its mRNA, reflecting a >3-fold reduction in Claspin’s half-life. Claspin contains a phosphodegron recognized by SCF^βTrCP^ E3 ubiquitin ligase when phosphorylated at Ser30/Ser34, a process involving PLK1 kinase. In PDAC cells, Claspin degradation upon Rac1 inhibition required the proteasome and βTrCP1/2 proteins, and was blocked by the mutagenesis of Ser30/Ser34, but occurred independently of PLK1 activity. Although Rac1 inhibitors reduced Claspin in both normal and cancer cells, PDAC cells may be uniquely vulnerable due to elevated replication stress and greater reliance on ATR/Chk1. Accordingly, Claspin depletion sensitized PDAC cells but not normal cells to gamma rays, inducing apoptosis only in cancer cells. Conclusions: These findings identify Rac1 as a critical regulator of ATR/Chk1 signaling through stabilization of the fork protection protein Claspin. Rac1 inhibition promotes the βTrCP-dependent, proteasome-mediated degradation of Claspin via its phosphodegron, thereby impairing Chk1 activation in response to DNA damage.

## Simple Summary

Pancreatic ductal adenocarcinoma (PDAC) is highly resistant to DNA-damaging therapies and is driven by oncogenic *KRAS* signaling. We found that inhibition of the *KRAS* effector Rac1 disrupts ATR/Chk1 DNA damage signaling by promoting the degradation of Claspin, a fork protection protein required for Chk1 activation. In PDAC and normal pancreatic ductal cells, Rac1 inhibition reduced Claspin protein stability without altering its mRNA levels, shortening its protein half-life more than threefold. Claspin loss required proteasomal degradation and βTrCP1/2, and depended on the Ser30/Ser34 phosphodegron, but occurred independently of PLK1 activity. Although Rac1 inhibition decreased Claspin in both normal and cancer cells, PDAC cells were selectively sensitized to γ-radiation, with Claspin depletion triggering apoptosis only in the tumor cells. These findings identify Rac1 as a key regulator of the replication stress response and suggest that targeting Rac1-mediated Claspin stabilization may enhance PDAC sensitivity to radiation therapy.

## 1. Introduction

Rac1 is a small GTPase that belongs to the Rho family of Ras GTPases [1,2]. Like other Rho GTPases, Rac1 functions as a signaling switch, relaying signals from membrane receptors to downstream pathways in the cytoplasm and nucleus. Rac1, like its Rho family counterparts, is isoprenylated—a post-translational modification that covalently attaches a lipid moiety to its C-terminal CAAX motif, specifically a geranylgeranyl group in the case of Rac1 [3,4]. This modification facilitates Rac1′s association with the plasma membrane, allowing it to interact with its regulators and effectors. Like other small GTPases, Rac1 cycles between an active GTP-bound state and an inactive GDP-bound state, with the balance between these two forms regulated by guanine nucleotide exchange factors (GEFs) and GTPase-activating proteins (GAPs). Rac1-specific GEFs catalyze the exchange of GDP for GTP, thereby activating Rac1 and enabling interactions with its downstream effectors. Rac1-specific GAPs stimulate the GTPase activity of Rac1 to promote the cleavage of GTP to GDP, thereby inactivating Rac1. At the plasma membrane, Rac1 plays a crucial role in remodeling the actin cytoskeleton, which gives cells their shape and enables the formation of lamellipodia and phagocytic cups, thereby supporting cell migration and macropinocytosis [1,2]. In many cancers, including pancreatic ductal adenocarcinoma (PDAC), Rac1 overexpression is associated with poor patient prognosis (TCGA database) [5]. Essential for the in vitro transformation of primary cells by oncogenic *KRAS* [6], Rac1 is also necessary for the development of Ras-driven lung and pancreatic cancers [7,8]. Rac1 also acts as a downstream effector of oncogenic *KRAS*, promoting migration and therapeutic resistance. Several small-molecule Rac1 inhibitors have been developed and tested in vivo, with some exhibiting encouraging antitumor activity in mouse models of lung, breast, brain, prostate, and colorectal cancers [9,10,11,12,13,14,15,16,17,18].

Mounting evidence implicates Rac1 as a key regulator of the DNA damage response (DDR). In many cell lines, active GTP-bound Rac1 is induced after exposure to gamma rays or other DNA-damaging agents [19,20,21]. Inhibiting or silencing Rac1 attenuates the DDR triggered by gamma rays [20,22,23] and UV light [20,21], sensitizing cells to these agents [19,20,22,23,24]. The DDR is orchestrated by the ATM and ATR kinases, along with their respective effector kinases, Chk2 and Chk1 [25,26]. These kinases are swiftly activated by DNA damage, coordinating the response. However, in cells treated with Rac1 inhibitors, ATM/Chk2 and ATR/Chk1 signaling are impaired, preventing the activation of these kinases following gamma-ray exposure [22,23]. This disruption abrogates the G2/M cell cycle checkpoint, allowing cells to enter mitosis with damaged DNA, leading to increased apoptosis and decreased survival [22,23]. Similar effects were observed in Rac1-deficient keratinocytes and HeLa cells, which also exhibited an attenuated DDR upon UV irradiation [20,21].

Rac1′s involvement in the DDR may be attributed to its nuclear fraction. Unlike most Rho GTPases, Rac1 is also detected in the nucleus, as shown using GFP-Rac1 fusions [27,28,29,30,31] and cell fractionation methods [30]. A triproline-polybasic region adjacent to the CAAX motif acts as a nuclear localization signal (NLS) and is required for nuclear localization [29,30,31]. Nuclear Rac1 appears to be isoprenylated, active, and loaded with GTP [30,32]. Its nuclear concentration peaks during the S and G2 phases of the cell cycle, increasing further after DNA damage [5,30]. Expression of a dominant-negative mutant of Rac1 (Rac1^T17N^) with a mutation that restricts its localization to the nucleus could partially inhibit the DDR induced by doxorubicin [33]. Continuous inhibition of Rac1 causes cancer cells to arrest in late S or G2 phases [34], a finding consistent with a role in ATR/Chk1 signaling and S phase progression.

In PDAC cells, the G1/S cell cycle checkpoint is often compromised due to genetic lesions in *KRAS*, *CDKN2A*, and/or *TP53*, making these cells more dependent on ATR/Chk1 signaling and intra-S checkpoint for survival. The ATR kinase is activated by DNA lesions containing single-strand DNA (ssDNA) regions, which occur during replicative stress [35,36,37]. ATR can also be activated by gamma rays following the processing of double-stranded DNA (dsDNA) breaks into lesions with ssDNA overhangs. ATR activation is a complex, multi-step process involving many factors (ATRIP, RPA, TopBP1, Rad17, RFC complex, and 9-1-1 complex) [35,36,37]. The process begins with RPA recognizing the ssDNA segment, forming a nucleofilament (step 1). The Rad9-Rad1-Hus1 clamp (9-1-1 complex) then identifies the ss/ds-DNA junction (step 2), while ATR-ATRIP recognizes the RPA nucleofilament (step 3). TopBP1 bridges the 9-1-1 clamp with ATR-ATRIP, leading to ATR activation (step 5). ATR can then phosphorylate itself and several other substrates but not yet its effector kinase, Chk1. The phosphorylation of Chk1 by ATR requires Claspin and other subunits of the Fork Protection Complex (FPC; step 6). Once activated, ATR and Chk1 act conjointly to phosphorylate cell cycle regulators, such as p53 and Cdc25, as well as factors involved in replication fork repair and restart (step 7). Once phosphorylated, transcription factor p53 induces expression of the cyclin-dependent kinase inhibitor p21^WAF1^, leading to cell cycle arrest. Cdc25 is a protein phosphatase that removes inhibitory phosphorylation from cyclin-dependent kinases (CDKs), and its phosphorylation and inhibition by ATR block the cell cycle. The ATR/Chk1 cascade operates during the S and G2 phases, and its disruption sensitizes cancer cells to replication stress and DNA damage.

A key component of the ATR/Chk1 cascade is the Claspin protein. Originally identified in Xenopus oocyte extracts, Claspin binds to Chk1 and is required for its phosphorylation by ATR [38]. Claspin functions by being both a subunit of the FPC and a bona fide component of the ATR/Chk1 cascade. The FPC, which includes proteins such as Tipin, Timeless, AND-1, FRAC, and Claspin [38,39], ensures that stalled replication forks remain intact. As part of the FPC, Claspin helps stabilize stalled replication forks by preventing their collapse. In the absence of the FPC, helicase and polymerase activities dissociate at stalled forks, causing excessive ssDNA accumulation and fork destabilization. The FPC, in part through Claspin, inhibits the CMG helicase (Cdc45-MCM-GINS) and Cdc7 kinase to prevent fork collapse [38,40,41]. Claspin further stabilizes the replisome through interactions with DNA polymerases and the PCNA clamp [42]. Claspin also guards against the effects of replication stress by being a key component of the ATR/Chk1 pathway. Claspin has two DNA-binding domains at its N-terminus, both with a preference for branched or forked DNA structures [43]. It has been proposed that Claspin uses these domains to detect ssDNA patches at stalled forks prior to inhibiting the CMG helicase and Cdc7 kinase. As a component of the FPC, Claspin would be ideally located to both sense the ssDNA patches at stalled forks and respond to them both by inhibiting the CMG helicase and by promoting ATR/Chk1 signaling. In the absence of Claspin, ATR/Chk1 signaling is inhibited, and cells become sensitized to drugs and conditions that cause replication stress [44,45,46,47].

Claspin levels fluctuate throughout the cell cycle, peaking in the S and G2 phases when replication stress is most likely to occur. This regulation is mediated by the activities of two E3 ubiquitin ligases, APC/C^Cdh1^ and SCF^ꞵTrCP^ [48,49,50,51,52,53,54]. These enzymes catalyze the poly-ubiquitination of Claspin, leading to its recognition by the 26S proteasome and its proteolytic degradation. APC/C^Cdh1^, which operates from the metaphase–anaphase transition to the end of the G1 phase, utilizes its Cdh1 subunit to recognize an LLK motif (aa 333–335) in Claspin [54]. SCF^βTrCP^ instead recognizes a phosphodegron located at the N-terminus of Claspin (DSGQGS; aa29-aa34). Recognition of this motif by the βTrCP1/2 subunits of SCF^βTrCP^ requires phosphorylation of the degron’s two serines (Ser30, Ser34). This phosphorylation occurs at the G2/M border of the cell cycle or after recovery from intra-S checkpoint activation, leading to Claspin ubiquitination and degradation. Together, SCF^βTrCP^ and APC/C^Cdh1^ ligases restrict Claspin protein expression to the S and G2 phases when it is most needed to mitigate the impacts of replication stress [48,49,50,51,52,53].

We previously reported that in PDAC cells treated with Rac1 inhibitors, ATR/Chk1 signaling is impeded and Chk1 fails to be activated by gamma rays [22,23]. This failure to activate Chk1 abolishes the G2/M checkpoint, allowing cells to proceed to mitosis with damaged DNA, leading to increased apoptosis and reduced survival [22,23]. In this article, we demonstrate that the loss of Claspin protein is responsible for this failure of Rac1-inhibited PDAC cells to activate Chk1 in response to gamma rays. Our results show that the Claspin protein is degraded once the activity of Rac1 is inhibited in PDAC cell lines, normal pancreatic ductal cells, and even human kidney embryonic HEK293T cells. Investigations into the implicated mechanisms have identified the SCF^βTrCP^ E3 ubiquitin ligase complex as the primary Claspin regulator affected by Rac1 activity. These results highlight the role of Rac1 as an upstream regulator of Claspin. The potential mechanisms involved and the implications of these findings for PDAC therapy are discussed.

## 2. Materials and Methods

### 2.1. Materials

Fetal bovine serum (FBS) was obtained from Atlas Biologicals (Fort Collins, CO, USA), and Medium M3 (cat# M3:BaseF) was purchased from InCell Corp. (San Antonio, TX, USA). Cycloheximide and the mammalian protease inhibitor cocktail were obtained from Sigma-Aldrich (Saint-Louis, MO, USA), whereas Volasertib (cat# S2235), EHT-1864 (cat# S7482) and NSC23766 (cat# S8031) were from Selleck Chemicals (Houston, TX, USA). AZA1 and MG132 were from MedChemExpress (Monmouth Junction, NJ, USA; HY-136383) and Enzo Life Sciences (Farmingdale, NY, USA; cat# BML-PI102), respectively. All other chemicals were purchased from Fisher Scientific (Waltham, MA, USA).

### 2.2. Cell Lines

The AsPC1, Panc1, HPAF/CD18, and L3.6pl cells used in the experiments were authenticated by STR profiling performed by Genetica, LabCorp (Burlington, NC, USA). HT1080 and HEK293T cells were gifts from Jerry Shay (UTSW Medical Center, Dallas, TX, USA). These cell lines were cultivated in DMEM media supplemented with 10% FBS and 50 μg/mL Gentamycin. hTERT-HPNE cells (referred to therein as HPNE cells) are a line of human primary pancreatic ductal cells immortalized by us using telomerase [55,56]. HPNE cells were cultivated in medium D, as described before [56]. All cells were cultivated at 37 °C in a humidified atmosphere containing 5% CO_2_.

### 2.3. siRNA Knockdowns

Cells were reverse-transfected with siRNA SMARTpools purchased from Horizon Discovery (Lafayette, CO, USA) and transfected using DharmaFECT1 (cat# T-2001) following the manufacturer’s instructions. The SMARTpools purchased included a non-targeting pool (cat# D-001810-10) and pools designed to target Claspin (cat# L-005288), βTrCP1 (cat# L-003463), βTrCP2 (cat# L-003490), or Rac1 (cat# L-003560) transcripts.

### 2.4. Western Blot Analysis

Samples were harvested in Laemmli buffer, as we have done before [57,58], with equal volumes of each sample analyzed and Ponceau S staining to confirm equal loading and transfer. Antibodies against GAPDH (cat# sc-47724), β-actin (cat# sc-1616), pATR (T1989) (cat# sc-58014S), and ATR (cat# sc-515173) were from Santa Cruz Biotechnology (Dallas, TX, USA). Antibodies against cleaved caspase 3 (clone 5A1E), Claspin (cat# 2800), pChk1 (S345) (cat# 2348), Chk1 (cat# 2G1D5), Rad9A (D2J4P), and TopBP1 (D8G4L) were purchased from Cell Signaling Technology (Danvers, MA, USA). Rac1 (23A8) antibody was purchased from Sigma Millipore (St. Louis, MO, USA). The secondary antibodies used were horseradish peroxidase-conjugated goat antibodies against mouse, rabbit, or rat IgG (Jackson ImmunoResearch, West Grove, PA, USA). The size markers used were the Precision Plus Protein TM Dual Color Standards (cat# 1610374) from Bio-Rad (Hercules, CA, USA). All uncropped blots (whole Western blots) are provided in the Supplementary file, along with their molecular weight markers.

### 2.5. Statistical Analyses of Western Blot Data

Experiments were performed independently on two separate occasions. Band intensities were quantified using ImageJ2 (version 1.54s) and normalized to their corresponding GAPDH or actin loading control for each lane. To enable comparison between independent experiments, the resulting normalized values were expressed as a fraction of the total signal across all lanes within each experiment. This normalization approach generated two independent biological replicates that were used for statistical analysis. Pairwise comparisons between treated and untreated groups were performed using two-tailed Student’s *t*-tests. No correction for multiple comparisons was applied.

### 2.6. Measuring Protein Stability

Claspin protein half-life was determined using the cycloheximide chase assay [59], as we have previously done for other proteins [58]. Experimental details are provided in the figure legend.

### 2.7. mRNA Quantitation by Real-Time RT-PCR

The Claspin and GAPDH mRNA transcripts were quantified by real-time qPCR using TaqMan Gene Expression Assays with FAM-conjugated minor groove binder (MGB) probes, as we have previously done for other transcripts [58]. The MGB probes used were designed to detect Claspin (cat# Hs00898637_m1) or GAPDH (cat# Hs99999905_m1) transcripts (ThermoFisher Sci., Waltham, MA, USA). Experimental details are provided in the figure legend.

### 2.8. Exposure to Gamma Rays

Exponentially growing cells were either mock-irradiated (0 Gy) or exposed to gamma rays in a Mark I 68A Cesium-137 Irradiator (10 Gy). For experiments involving treatment with both NSC23766 and gamma rays, cells were incubated with NSC23766 for 1 h prior to receiving their dose of gamma rays.

### 2.9. FLAG-Claspin Expression Vectors

Mammalian expression vector pcDNA3.1-FLAG-Claspin was a gift from Michele Pagano (Addgene plasmid # 12659). This vector encoding wild-type (wt) Claspin was mutagenized using the QuikChange II XL Site-Directed Mutagenesis Kit (Agilent, Santa Clara, USA) to create its S30A/S34A mutant (mut). Oligonucleotides used for mutagenesis had the following sequences: 5′-GATAGTCCTTCAGATGCTGGACAGGGCGCC-TATGAAACAATTGG-3′ (top strand; mutated nucleotides underlined) and 5′-CCAATTGTTTCATAGGCGCCCTGTCCAGCATCTGAAGGACTATC-3′ (bottom strand; mutated nucleotides underlined).

### 2.10. Transfection of HEK293T Cells

In duplicates, exponentially growing HEK293T cells were transfected in 6-well plates with Lipofectamine 2000 reagent following the manufacturer’s instructions (ThermoFisher Sci., Waltham, MA, USA). In experiments involving NSC23766, cells were transfected 2 days prior to being exposed to either NSC23766 (100 μM) or vehicle (DMSO).

## 3. Results

### 3.1. Rac1 Inhibition Blocks the S345-Phosphorylation of Chk1 by ATR

**Figure 1:**
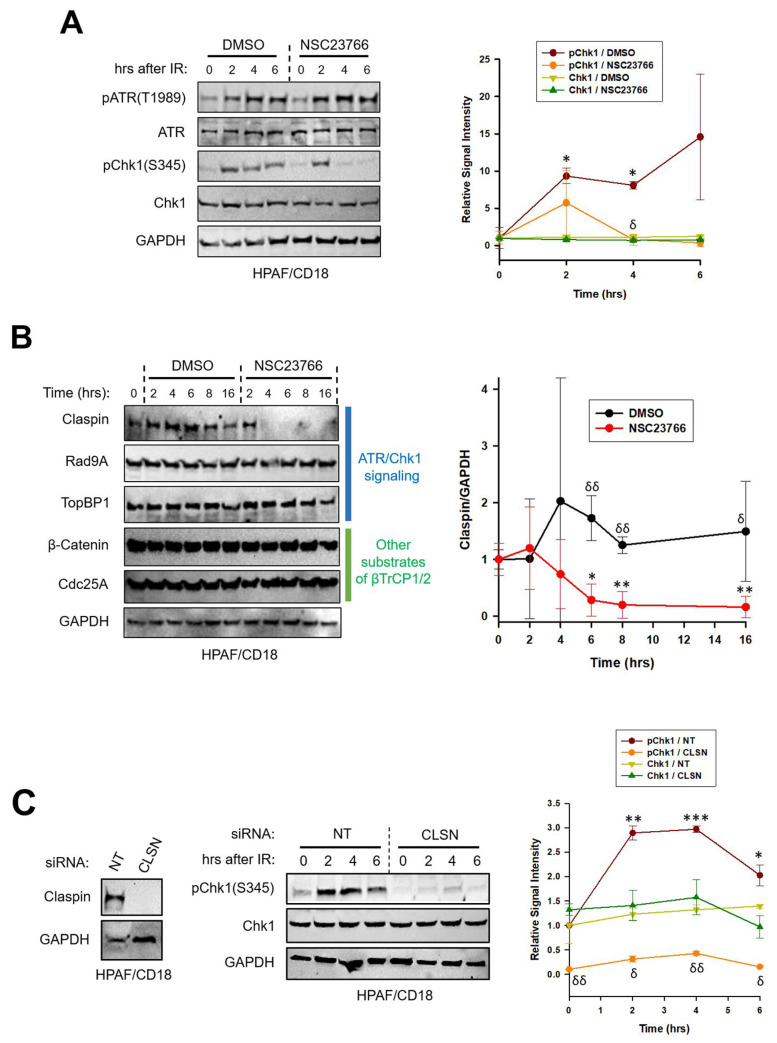
The loss of Claspin protein is responsible for the failure of Rac1-inibited PDAC cells to induce the S345-phosphorylation in response to gamma rays. (**A**) Exposure to Rac1 inhibitor NSC23766 reduces the ability of HPAF/CD18 cells to phosphorylate Chk1 in response to ionizing radiation (IR). Cells were pre-treated with NSC23766 (100 μM) or vehicle (DMSO) before being exposed to a single dose of gamma rays (10 Gy). Duplicate series of samples were harvested at the indicated times post-irradiation and probed with the indicated antibodies. GAPDH was used as an internal standard. The graph shows the signal intensities of Chk1 and pChk1 over time, expressed as mean ± S.D. (n = 2). Statistically different from time 0 in Student’s *t* test (*, *p* < 0.05). Statistically different from DMSO in Student’s *t* test (δ, *p* < 0.01). (**B**) Exposure to NSC23766 reduces Claspin levels in HPAF/CD18 cells. Cells were exposed to NSC23766 (100 μM; n = 4) or vehicle (DMSO; n = 2) for the indicated time before being harvested and analyzed for the presence of Claspin, markers of ATR/Chk1 signaling, and different known substrates of SCF^βTrCP1/2^. GAPDH was used as an invariant internal control. The graph shows the Claspin/GAPDH ratio over time, expressed as mean ± S.D. (n = 2–4). Statistically different from time 0 in Student’s *t* test (*, *p* < 0.05; **, *p* < 0.01). Statistically different from NSC23766 in Student’s *t* test (δ, *p* < 0.05, δδ, *p* < 0.01). (**C**) Claspin depletion blocks the ability of HPAF/CD18 cells to phosphorylate Chk1 in response to ionizing radiation (IR). Duplicate series of HPAF/CD18 cells were transfected with either Claspin siRNAs (CLSN) or a non-targeting siRNA (NT) as a control. Two days post-transfection, cells were analyzed for differences in Claspin levels (left panel) and then exposed to a single dose of gamma rays (10 Gy), after which samples were collected at the indicated time points (right panel). GAPDH was used as an internal standard. Graph shows the signal intensities of Chk1 and pChk1 over time, expressed as mean ± S.D. (n = 2). Statistically different from time 0 in Student’s *t* test (*, *p* < 0.05; **, *p* < 0.01; ***, *p* < 0.001). Statistically different from NT in Student’s *t* test (δ, *p* < 0.01; δδ, *p* < 0.001).

We previously identified Rac1 as a novel regulator of the DNA damage response (DDR). In breast and pancreatic cancer cell lines, we reported that Rac1 inhibition disrupts activation of the ATR/Chk1 and ATM/Chk2 cascades after gamma-ray exposure, thereby resulting in radiosensitivity [22,23]. In this article, we are investigating the mechanism by which Rac1 regulates activation of the ATR/Chk1 cascade. To pinpoint the step(s) of ATR activation influenced by Rac1, we used phospho-specific antibodies to investigate the phosphorylation of ATR and Chk1 (Figure 1A). These experiments were conducted in HPAF/CD18 cells, a radioresistant pancreatic ductal adenocarcinoma (PDAC) cell line with oncogenic *KRAS* expression and elevated Rac1 activity [23]. Cells were pretreated with Rac1 inhibitor NSC23766 (100 μM) or vehicle control (DMSO) for one hour, followed by a single dose of gamma rays (10 Gy). Duplicate samples were collected at various times post-irradiation. In vehicle-treated cells, γ-irradiation induced a rapid increase in ATR phosphorylation at threonine 1989 (T1989) and Chk1 phosphorylation at serine 345 (S345) within two hours (Figure 1A). In contrast, while ATR T1989 phosphorylation was still observed in the NSC23766-treated cells, Chk1 S345 phosphorylation was greatly reduced and limited to the 2 h mark (Figure 1A). These results show that Rac1 inhibition selectively disrupts the S345 phosphorylation of Chk1 by ATR, a process known to depend on the adaptor protein Claspin [25,42,60,61].

### 3.2. Rac1 Inhibition or Depletion Reduces Claspin Protein Levels

The phosphorylation of Chk1 at serine 345 (S345) by ATR requires Claspin, an adaptor protein that facilitates the interaction between Chk1 and ATR [38,42,62]. To assess the role of Rac1 in regulating Claspin and other components of the ATR signaling pathway, we examined protein levels of Claspin, Rad9A, and TopBP1 in HPAF/CD18 cells following Rac1 inhibition. Cells were treated with either NSC23766 (100 μM) or vehicle (DMSO), and samples were collected at various time points. Treatment with NSC23766 caused a five- to six-fold reduction in Claspin protein within 8 h of exposure (Figure 1B). In contrast, Claspin levels remained statistically unchanged in the DMSO-treated control cells. The levels of Rad9A and TopBP1 were unaffected by either treatment, as were other proteins such as β-catenin and Cdc25A. These results suggest that Rac1 inhibition specifically impacts Claspin without broadly altering other components of the ATR signaling machinery.

To determine whether Claspin depletion alone can account for the impaired ATR/Chk1 signaling observed after Rac1 inhibition, we silenced Claspin in HPAF/CD18 cells using siRNA. Two days later, cells were irradiated with a single dose of gamma rays (0 or 10 Gy) and assessed for ATR/Chk1 pathway activation (Figure 1C). In control cells transfected with non-targeting siRNA, γ-irradiation led to a three-fold increase in Chk1 S345 phosphorylation within 2 h. However, this phosphorylation event was absent in cells transfected with Claspin siRNA (Figure 1C), thereby confirming that Claspin is essential for ATR-mediated Chk1 activation.

**Figure 2:**
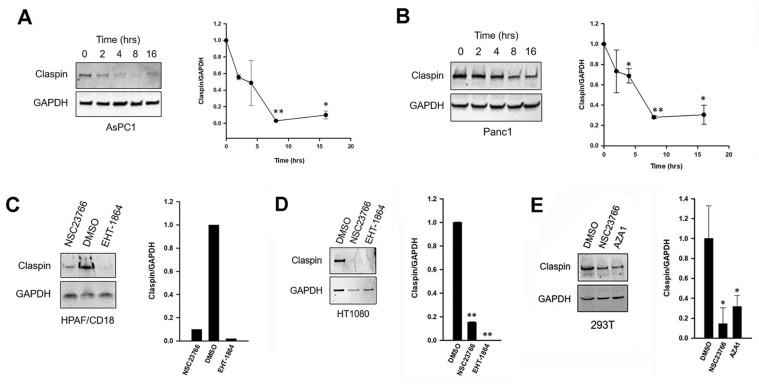
Rac1 inhibitors reduces Claspin levels in a wide range of cancer cells lines. Claspin levels in AsPC1 (**A**), Panc1 (**B**) cells after NSC23766 treatment. In duplicate series of samples, the indicated PDAC lines were exposed to NSC23766 (100 μM) and harvested at the indicated time points. GAPDH was used as an internal standard. The graph shows the Claspin/GAPDH ratio over time, expressed as mean ± S.D. (n = 2). Statistically different from time 0 in Student’s *t* test (*, *p* < 0.05; **, *p* < 0.01). Claspin levels in HPAF/CD18 (**C**) (n = 1), HT1080 (**D**) (n = 2), and HEK293T (**E**) (n = 2) cells exposed to different Rac1 inhibitors. Claspin was quantified after 16 h of exposure to NSC23766 (100 μM), EHT-1864 (50 μM), AZA1 (20 μM), or DMSO vehicle. GAPDH was used as an internal control. The bar graph to the right show the Claspin/GAPDH ratio. Statistically different from DMSO in Student’s *t* test (*, *p* < 0.05; **, *p* < 0.001).

To assess whether Rac1 regulates Claspin expression across additional cell lines, we expanded our analysis to additional PDAC cell lines, including AsPc1 (Figure 2A) and Panc1 cells (Figure 2B). In both lines, treatment with NSC23766 produced a four- to five-fold reduction in Claspin protein levels. Claspin expression was likewise decreased by the Rac1 inhibitor EHT-1864, which is structurally distinct from NSC23766 and inhibits Rac1 through a different mechanism [63,64]. In HPAF/CD18 cells, EHT-1864 was even more effective than NSC23766 in lowering Claspin levels (Figure 2C). The Rac1 inhibitors also reduced Claspin in neoplastic lines derived and established from other organs, including the prostate (HT1080 cells; Figure 2D) and kidney (HEK293T cells; Figure 2E).

**Figure 3:**
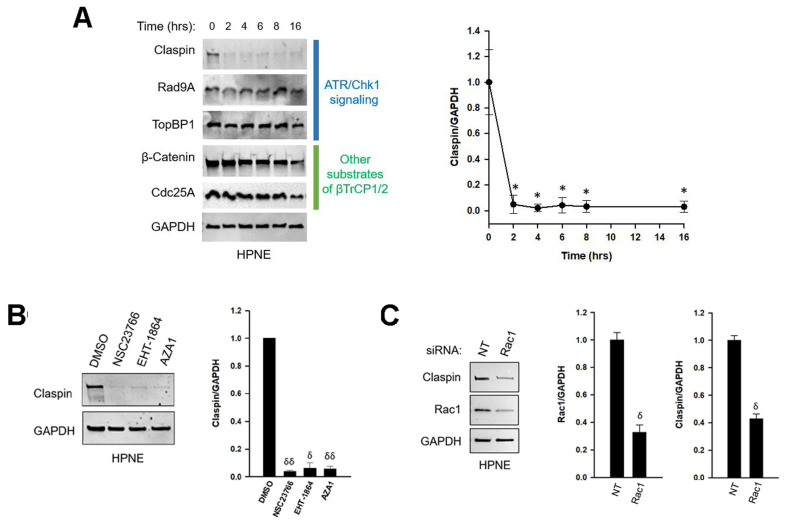
Rac1 inhibitors reduce Claspin levels in normal human pancreatic ductal cells. (**A**) Exposure to NSC23766 reduces Claspin levels in HPNE cells. In duplicate series of samples, HPNE cells were exposed to NSC23766 (100 μM) for the indicated time before being analyzed for the presence of Claspin, markers of ATR/Chk1 signaling, and known substrates of SCF^βTrCP1/2^. GAPDH was used as an internal control. The graph shows the Claspin/GAPDH ratio over time, expressed as mean ± S.D. (n = 2). Statistically different from time 0 in Student’s *t* test (*, *p* < 0.05). (**B**) Claspin levels in HPNE cells exposed to different Rac1 inhibitors. In duplicates, HPNE cells were exposed to NSC23766 (100 μM), EHT-1864 (50 μM), or AZA1 (20 μM). Sixteen hours later, cells were analyzed for Claspin levels. The bar graph shows the Claspin/GAPDH ratio as mean ± S.D. (n = 2). Statistically different from DMSO in Student’s *t* test (δ, *p* < 0.01; δδ, *p* < 0.001). (**C**) The siRNA-mediated silencing of Rac1 reduces Claspin levels in pancreatic cells. In duplicates, HPNE cells were transfected with either Rac1 siRNAs (Rac1) or a non-targeting siRNA (NT). Two days later, the levels of Claspin and Rac1 were measured by Western blotting. GAPDH was used as an internal standard. The bar graphs show the Rac1/GAPDH ratios and Claspin/GAPDH ratios as mean ± S.D. (n = 2). Statistically different from the NT-transfected cells in Student’s *t* test (δ, *p* < 0.01).

Next, we assessed whether Rac1 inhibition triggered similar changes in normal human cells, using hTERT-HPNE cells as a model system (therein termed HPNE). HPNE cells are human primary pancreatic ductal cells immortalized by us using telomerase [55,56]. In HPNE cells, NSC23766 led to a 10-fold decline in Claspin levels within 2 h of exposure (Figure 3A). As in the HPAF/CD18 cells (Figure 1B), NSC23766 caused Claspin levels to decline with no alterations in the levels of either TopBP1 or Rad9A (Figure 3A). This decline in Claspin level could also be elicited by other Rac1 inhibitors (Figure 3B), including EHT-1864 [63,64] and AZA1 [12]. In HPNE cells, Claspin levels could also be reduced by the transfection of siRNA directed against Rac1. HPNE cells were transfected with Rac1 siRNA or a non-targeting siRNA. Two days later, cells were analyzed for differences in Claspin levels. In HPNE cells transfected with Rac1 siRNA, levels of Rac1 and Claspin were reduced by 67% and 57%, respectively (Figure 3C). These findings demonstrate that Rac1 regulates Claspin expression in a variety of human pancreatic cell lines, both cancerous and normal.

### 3.3. Rac1 Regulates Claspin Protein Stability

**Figure 4:**
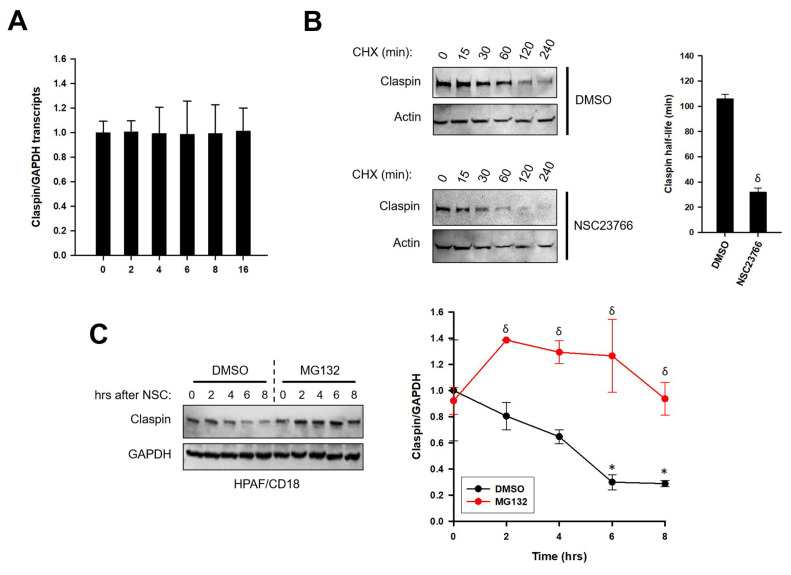
Rac1 inhibition reduces Claspin protein stability. (**A**) Rac1 inhibition has no impact on the abundance of the Claspin mRNA. HPAF/CD18 cells were harvested at the indicated times after NSC23766 (100 μM), after which RNA was isolated. Real-time qRT-PCR was then used to quantify the Claspin mRNA (graph). Claspin/GAPDH mRNA ratios are displayed as mean ± S.D. from triplicate samples (n = 3). (**B**) Rac1 inhibition reduces Claspin stability in HPAF/CD18 cells. In duplicate series of samples, cells were treated with NSC23766 (100 μM) or vehicle (DMSO). After 2 h of exposure, cycloheximide was added to block protein synthesis (50 μg/mL), after which samples were harvested at the indicated times to monitor Claspin. Actin was used as an internal control. The bar graph displays Claspin’s calculated half-lives under Rac1-proficient and -inhibited conditions, quantified using ImageJ (version 1.54s) and fitted to an exponential decay curve to estimate Claspin’s half-lives, expressed as mean ± S.D. (n = 2). Statistically different from DMSO in Student’s *t* test (δ, *p* < 0.01). (**C**) Proteasome inhibitor MG132 prevents Claspin degradation induced by NSC23766. In duplicate series of samples, HPAF/CD18 cells were first exposed to MG132 (20 μM) or vehicle (DMSO). One hour later, NSC23766 (100 μM) was added, after which cells were harvested at the indicated time points and analyzed for Claspin levels. GAPDH was used as an internal standard. The graph shows the Claspin/GAPDH ratios over time, expressed as mean ± S.D. (n = 2). Statistically different from time 0 in Student’s *t* test (*, *p* < 0.05). Statistically different from DMSO in Student’s *t* test (δ, *p* < 0.05).

To assess whether Rac1 regulates Claspin at the mRNA levels, we used real-time qPCR to quantify the Claspin mRNA in HPAF/CD18 cells treated with NCS23766. Although NSC23766 caused the Claspin protein to decline, there were no changes in the level of the Claspin mRNA (Figure 4A). To test the possibility that Rac1 regulates Claspin at the level of protein stability, we used a cycloheximide chase assay [59] to measure the half-life of Claspin in HPAF/CD18 cells treated with NSC23766 (Figure 4B). In duplicate sets, cells were treated with either 100 μM NSC23766 or vehicle control (DMSO) for 2 h, followed by the addition of cycloheximide to block new protein synthesis. Claspin protein levels were then measured at multiple time points to determine its degradation rate (Figure 4B; Western blots). In vehicle-treated cells, Claspin levels gradually decreased, with a calculated half-life of 106 ± 3 min (n = 2) (Figure 4B; graph). In contrast, NSC23766 treatment significantly accelerated Claspin degradation, reducing its half-life to 32 ± 3 min (n = 2). This difference was statistically significant (Student’s *t*-test, *p* < 0.01). These findings demonstrate that Rac1 activity contributes to the stabilization of Claspin protein in HPAF/CD18 cells. Next, we investigate whether the ubiquitin-proteasome system (UPS) is involved in mediating Claspin degradation after Rac1 inhibition. We treated HPAF/CD18 cells with the proteasome inhibitor MG132 (20 μM) or vehicle (DMSO) for one hour prior to adding the Rac1 inhibitor NSC23766. Western blot analysis showed that MG132 pre-treatment blocked the NSC23766-induced reduction in Claspin levels, indicating that Claspin degradation occurs through the UPS (Figure 4C).

### 3.4. Claspin Degradation After Rac1 Inhibition Is Mediated by the SCF^βTrCP^ E3 Ubiquitin Ligase

**Figure 5:**
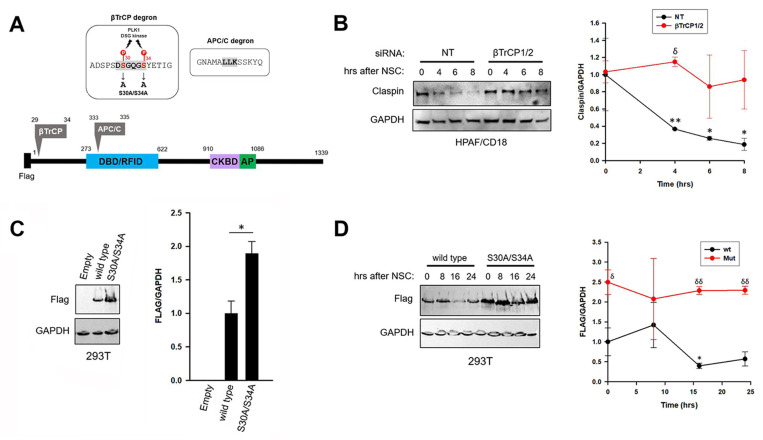
The SCF^βTrCP^ E3 ubiquitin ligase is required for Claspin protein degradation induced by NSC23766. (**A**) The βTrCP degron of Claspin, its phosphorylation, and its mutagenesis. The Claspin protein possesses at least two degrons that control its protein stability: a βTrCP degron (aa 29–34) and a APC/C degron (aa 333–335). Activation of the βTrCP degron and its recognition by the SCF^βTrCP^ E3 ubiquitin ligase requires the phosphorylation of its serines Ser30 and Ser34, an event catalyzed by the indicated kinases. Recognition of the APC/C degron by APC/C relies on its LLK motif and does not require phosphorylation. (**B**) The depletion of the βTrCP1/2 proteins blocks Claspin degradation induced by NSC23766. In duplicate series of samples, HPAF/CD18 cells were co-transfected with either βTrCP1 and βTrCP2 siRNAs (βTrCP1/2) or with a non-targeting siRNA (NT). Two days post-transfection, cells were exposed to NSC23766 (100 μM) and samples were collected at the indicated time points and analyzed for Claspin levels. GAPDH was used as an invariant internal control. The graph shows the Claspin/GAPDH ratios over time, expressed as mean ± S.D. (n = 2). Statistically different from time 0 in Student’s *t* test (*, *p* < 0.05; **, *p* < 0.01). Statistically different from NT in Student’s *t* test (δ, *p* < 0.01). (**C**) Detection of the FLAG-tagged Claspin proteins in transfected HEK293T cells. Cells were transfected with plasmids carrying no insert or expressing Flag-tagged Claspin, either the wild-type protein or its S30A/S34A mutant. Two days later, cells were probed with antibodies against Claspin or the FLAG tag. GAPDH was used as an invariant internal control. The graph shows the Claspin/GAPDH ratios, expressed as mean ± S.D. (n = 2). Statistically different in a Student’s *t* test (*, *p* < 0.05). (**D**) The S30A/S34A mutation blocks the degradation of Claspin triggered by the inhibition of Rac1. In duplicate series of samples, HEK293T cells were transiently transfected with plasmids encoding FLAG-tagged version of wild-type Claspin or its S30A/S34A mutant. Two days later, cells were exposed to NSC23766 (100 μM), after which samples were collected at the indicated time points. Samples were probed with antibodies against the FLAG tag. GAPDH was used as an invariant internal control. The graph shows the FLAG/GAPDH ratios, expressed as mean ± S.D. (n = 2). Statistically different from time 0 in Student’s *t* test (*, *p* < 0.05). Statistically different from wild-type Claspin in Student’s *t* test (δ, *p* < 0.05; δδ, *p* < 0.01).

Claspin stability is regulated by at least two E3 ubiquitin ligases: SCF^βTrCP^ and APC/C^Cdh1^ (Figure 5A) [42,51,52,54]. SCF^βTrCP^ recognizes a phosphodegron at the N-terminus of Claspin (DSGxxS motif; amino acids 29–34), whereas APC/C^Cdh1^ targets an LLK box located at amino acids 332–334. To assess the role of SCF^βTrCP^ in Claspin degradation, we transfected HPAF/CD18 cells with siRNAs targeting βTrCP1 and βTrCP2, the substrate recognition subunits of the SCF^βTrCP^ complex. Two days later, cells were treated with the Rac1 inhibitor NSC23766 and analyzed by Western blotting (Figure 5B). In control cells transfected with non-targeting siRNA, Claspin levels declined rapidly following NSC23766 treatment. In contrast, Claspin degradation was blocked in cells lacking βTrCP1/2, indicating that SCF^βTrCP^ is required for Claspin destabilization under these conditions.

### 3.5. Claspin Degradation Following Rac1 Inhibition Requires an Intact βTrCP Degron

Recognition of Claspin by the SCF^βTrCP^ E3 ligase complex depends on the phosphorylation of serine residues at positions 30 and 34 within its βTrCP degron. To further confirm the involvement of SCF^βTrCP^ in Rac1-mediated regulation of Claspin, these two serines were mutated in a mammalian vector encoding a N-terminally FLAG-tagged human Claspin protein (FLAG-Claspin). Perhaps because of the low transfectability of PDAC cell lines and the large size of the Claspin vectors, neither the wild-type vector (WT) nor the mutant vector (S30A/S34A) produced detectable FLAG-tagged Claspin protein in any of the pancreatic cell lines tested. To address this limitation, we tested the vectors in HEK293T cells, which offer exceptionally high transfection efficiency and also exhibit Rac1-dependent regulation of Claspin (Figure 2E). Forty-eight hours after transfection, FLAG-Claspin proteins were readily detected in cells expressing either the wild-type or the S30A/S34A mutant constructs, but not in cells transfected with the empty control vector (Figure 5C). Notably, expression levels were higher in cells transfected with the mutant construct compared to the wild-type one, consistent with previous reports that the βTrCP degron regulates Claspin stability [51,52]. To directly assess the contribution of the βTrCP degron to Claspin regulation by Rac1, HEK293T cells transfected with two vectors were exposed to NSC23766 (Figure 5D). Consistent with the results of Figure 5C, Western blotting with an anti-FLAG antibody revealed that baseline levels of the mutant FLAG-Claspin protein were higher than those of the wild-type one. Importantly, following Rac1 inhibition, wild-type FLAG-Claspin levels declined rapidly, whereas the mutant protein remained stable over time (Figure 5D). These results show that the regulation of Claspin by Rac1 depends on the integrity of the βTrCP degron.

### 3.6. Claspin Depletion Cooperates with Gamma Rays to Induce Apoptosis in PDAC Cells but Not Normal Cells

**Figure 6:**
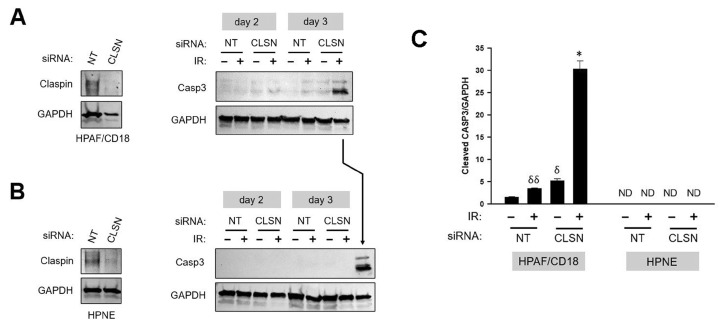
Claspin depletion cooperates with gamma rays to induce apoptosis in PDAC cells, but not normal cells. In duplicate series of samples, HPAF/CD18 (**A**) and hTERT-HPNE cells (**B**) were transfected with Claspin or non-targeting siRNA. Two days later, cells were exposed to ionizing radiation (IR) in the form of a single dose of gamma rays (0 or 10 Gy). On days 2 and 3 post-irradiation, cells were analyzed by Western blot for Claspin levels and the presence of cleaved Caspase 3. GAPDH was used as an invariant internal control. * Positive control made of Claspin-depleted γ-irradiated HPAF/CD18 cells, taken from Figure 6A. (**C**) Signal quantification of cleaved Caspase 3. The graph shows the cleaved CASP3/GAPDH ratios, expressed as mean ± S.D. (n = 2). Statistically different from all other groups (*, *p* < 0.01). Statistically different from untreated NT (δ, *p* < 0.05; δδ, *p* < 0.01). ND: not detected.

*KRAS*-driven cancers, such as PDAC, often exhibit high levels of replication stress and must adapt accordingly [65,66]. Our results show that Rac1 inhibition or depletion triggers Claspin degradation in both normal human pancreatic cells (Figure 3) and PDAC cells (Figure 1B and Figure 2A–D). However, due to their inherently higher levels of replicative stress, PDAC cells may be more reliant on Claspin for their survival and resistance to DNA damage. To test this hypothesis, we knocked down Claspin in HPAF/CD18 (Figure 6A) and HPNE cells (Figure 6B), after which we assessed their respective responses to γ-irradiation. After transfection with either the non-targeting or Claspin siRNA, duplicate sets of cells were exposed to a single dose of gamma rays (0 or 10 Gy). Two and three days post-irradiation, cells were examined for evidence of apoptosis, using cleaved Caspase 3 as a marker. In HPAF/CD18 cells, Claspin silencing cooperated with γ-irradiation to induce apoptosis (Figure 6A,C). By three days post-irradiation, apoptosis was most evident only in the Claspin-depleted, γ-irradiated cells, whereas γ-irradiation alone or Claspin knockdown alone produced minimal effects. In contrast, this cooperative effect between gamma rays and Claspin depletion was not observed in the normal HPNE cells (Figure 6B,C). In HPNE cells, neither Claspin silencing nor γ-irradiation or even their combined actions could induce apoptosis. These findings indicate that HPAF/CD18 cells are more effectively radiosensitized by Claspin depletion than the HPNE cells.

## 4. Discussion

At the plasma membrane, Rac1 interacts with its regulators and effectors to perform its function as a master regulator of the actin cytoskeleton, playing a key role in forming lamellipodia and macropinocytic cups, as well as facilitating cell migration. But mounting evidence has now also implicated Rac1 as a crucial regulator of the cellular response to DNA damage. In various forms of cancer, Rac1 inhibition or depletion consistently sensitizes cancer cells to DNA-damaging agents, including 5-fluorouracil [67], doxorubicin [33], UV light [20,21], and gamma rays [19,20,22,23]. In previous publications, we used an IP-kinase assay to monitor the activation of the ATM/Chk2 and ATR/Chk1 cascades after gamma rays [22,23] and reported that these kinases were no longer activatable in breast and pancreatic cancer cells treated with NSC23766 or expressing a dominant-negative mutant of Rac1 (Rac1^T17N^). This inhibition led to an abrogation of the G2/M cell cycle checkpoint, allowing cells with damaged DNA to enter mitosis, thereby causing apoptosis and reducing clonogenic survival [22,23]. This article examines the mechanisms underlying Rac1′s regulation of ATR/Chk1 signaling. With phospho-specific antibodies, we first noted that Rac1 inhibition blocked the S345-phosphorylation of Chk1 by ATR, but not the T1989-phosphorylation of ATR by itself (Figure 1A). The Claspin protein, essential for this phosphorylation event, was then found to decline in a time-dependent manner after Rac1 inhibition (Figure 1B). This decline was observed in three PDAC cell lines (HPAF/CD18, AsPC1, and Panc1) and in cancer cells of other origins (HT1080, HEK293T), as well as in normal pancreatic ductal cells (HPNE). It was also observed in cells treated with three different Rac1 inhibitors (NSC23766, EHT-1864, AZA1). Whereas NSC23766 and AZA1 are structurally related and work by blocking the interaction of Rac1 with its GEFs [11,68,69,70], EHT-1864 works differently and causes the loss of the bound nucleotide [63,64]. Although these pharmacological inhibitors are imperfect tools with their own off-target effects [71,72,73], they share the commonality of blocking the accumulation of active Rac1 [11,68,69,70,74]. Most importantly, Claspin levels were also reduced in HPNE cells by the siRNA-mediated silencing of Rac1 (Figure 3C). While we cannot exclude the involvement of other Rac1-related Rho GTPases, these observations unambiguously reveal Rac1 as a key regulator of Claspin levels in human cells. This down-regulation of Claspin explains, at least in part, the previously reported impacts of Rac1 inhibitors on ATR/Chk1 signaling. In line with this notion, the knockdown of Claspin was sufficient to abolish the radiation-induced S345-phosphorylation of Chk1, similar to the effects of Rac1 inhibition (Figure 1C). This novel regulation of Claspin by Rac1 could therefore account for the previously reported radio- and chemo-sensitizing activities of Rac1 inhibitors [19,20,21,22,23,33,67].

We investigated how Rac1 inhibition leads to a decline in Claspin protein levels and found that this effect is not due to changes in Claspin mRNA expression (Figure 4A). Instead, Rac1 inhibition significantly reduced Claspin protein stability, shortening its half-life from 106 ± 3 min to just 32 ± 3 min (Figure 4B). This destabilization was reversed by the proteasome inhibitor MG132, indicating that Claspin degradation occurs through the ubiquitin-proteasome system (UPS) (Figure 4C). The UPS is composed of more than 600 different E3 ubiquitin ligases, each with its own substrate specificity. Of these, two have been reported to mediate Claspin degradation: APC/C^Cdh1^ and SCF^ꞵTrCP^ (Figure 5A) [48,49,50,51,52,53]. Because APC/C activity and its downstream effects are primarily regulated by cell cycle events rather than by external stimuli [75,76], we first focused on SCF^βTrCP^. In contrast to APC/C, substrate recognition by SCF^βTrCP^ is typically controlled by signaling pathways that phosphorylate βTrCP degron motifs [77,78], making it a plausible mediator of Rac1-dependent regulation of Claspin stability. Furthermore, in our previous studies of PDAC cells, Rac1 inhibition did not alter cell cycle distribution, arguing against a direct influence on APC/C function itself [23]. We examined the role of SCF^ꞵTrCP^ by knocking down its substrate recognition subunits, βTrCP1 and βTrCP2. This intervention prevented the Rac1 inhibition-induced decrease in Claspin levels, confirming SCF^ꞵTrCP^’s involvement (Figure 5B). Claspin contains a known phosphodegron, the DSGxxS motif (amino acids 29–34), which must be phosphorylated at Ser30 and Ser34 for recognition by βTrCP1/2 (Figure 5A) [51,52,53]. To assess the functional importance of this motif, we mutated these two serines to alanines in a FLAG-tagged Claspin construct. In HEK293T cells, which also show Claspin downregulation upon Rac1 inhibition (Figure 2E), these mutations prevented the degradation of Claspin following Rac1 inhibition, further implicating the SCF^ꞵTrCP^ ligase (Figure 5C,D). Collectively, these findings demonstrate that Rac1 inhibition promotes Claspin degradation through the SCF^ꞵTrCP^ ligase, and that this process depends on a functional phosphodegron within Claspin.

**Figure 7:**
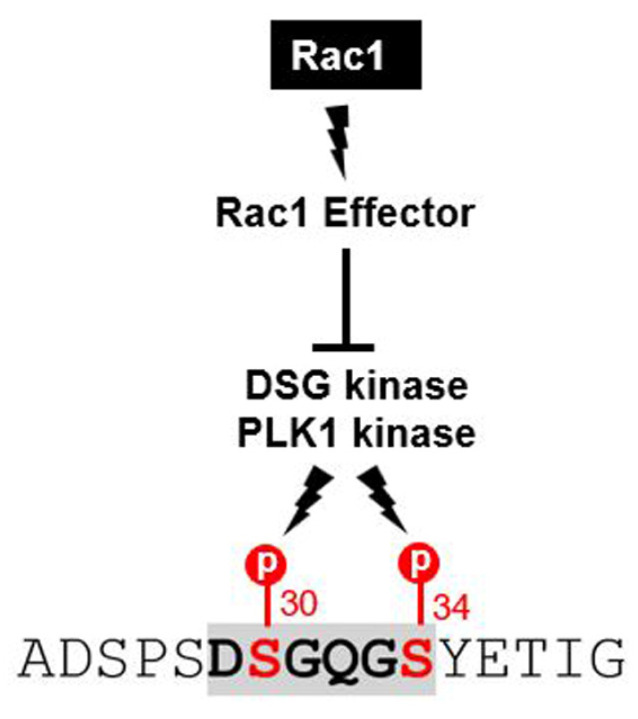
A model for the phosphorylation and degradation of Claspin in Rac1-inhibited cells. The Claspin protein carries a degron recognized by the SCFβTrCP E3 ubiquitin ligase when phosphorylated at its two serine residues (Ser30, Ser34). Based on our results, we propose that the recognition of this degron by the βTrCP1/2 proteins is co-regulated by PLK1 and a so-called “DSG” kinase that phosphorylate the S30 residues of Claspin. We propose that this kinase is regulated by the activity of a Rac1 effector that regulates the S30-phosphorylation of Claspin by a so-called “DSG” kinase independently of the PLK1 kinase.

Although PLK1 has been implicated in the regulation of Claspin degradation by the SCF^βTrCP^ ubiquitin ligase complex [51,52,53], the βTrCP degron within Claspin lacks a consensus PLK1 phosphorylation motif, suggesting the involvement of an alternative kinase. Supporting this interpretation, PLK1 inhibition increased basal Claspin levels but did not prevent Claspin degradation following inhibition of Rac1 (Figure S1), indicating that PLK1 is unlikely to mediate Rac1-dependent regulation of Claspin stability. In a recent study published in this journal, we described a similar Rac1-dependent mechanism controlling the stability of YAP1 protein, a transcriptional co-activator involved in pathways that sense cell–cell and cell–matrix interactions [57]. Similar to Claspin, YAP stability is controlled by Rac1, such that Rac1 inhibition promotes its βTrCP-mediated proteolysis. Importantly, activation of the βTrCP degron of YAP occurred independently of the canonical pathway, which for YAP involves its phosphorylation by the LATS1/2 kinases. Based on these and other findings, we had proposed a model in which the DSG motif of the βTrCP degron is directly phosphorylated by a putative “DSG kinase,” thereby triggering degron activation and YAP degradation [57]. This putative “DSG kinase” could potentially mediate the Rac1-dependent regulation of both YAP and Claspin through direct phosphorylation of the conserved DSG motif, followed by the secondary phosphorylation of downstream serine residues, thereby activating the βTrCP degron (Figure 7). Notably, this kinase, activated upon Rac1 inhibition, does not appear to regulate all βTrCP substrates, as other known substrates, such as CDC25A [79] and β-catenin [80], were unaffected (Figure 1B). These observations suggest that the kinase exhibits substrate selectivity, recognizing specific βTrCP degrons, such as those present in YAP and Claspin, while excluding others.

One unresolved question is how Rac1, a small GTPase primarily associated with the plasma membrane, can influence nuclear processes such as the DNA damage response (DDR). For Rac1 to regulate Claspin stability, a Rac1 effector must be able to transmit signals into the nucleus. An alternative and compelling possibility is that Rac1 itself mediates the nuclear signaling. Unlike most GTPases, Rac1 contains a nuclear localization signal and translocates to the nucleus during the S and G2 phases of the cell cycle, precisely when Claspin levels are highest [30,31,32,33]. In the nucleus, Rac1 may engage with a distinct set of effectors or regulatory proteins that influence the DDR and Claspin stability. Identifying these nuclear Rac1 effectors and understanding how they contribute to Claspin regulation and the radioresponse of PDAC cells remains an important direction for future studies.

This study sheds new light on how Rac1 inhibitors exert their radio- and chemo-sensitizing effects, offering a clearer understanding of their therapeutic potential. With this mechanistic understanding, it may become possible to identify which patients are most likely to benefit from Rac1-targeted therapies. Cancers driven by activated oncogenes, such as PDAC, often exhibit high levels of replication stress, and therapeutic strategies have been developed that take advantage of this vulnerability [25,65,66]. Claspin plays a critical role in helping cells manage this stress. It is frequently overexpressed in various cancers, where its elevated expression correlates with poor patient outcomes [42,45]. Because cancer cells endure higher replication stress than normal cells, they are more dependent on the ATR/Chk1 signaling pathway for their survival and proliferation [25,65,66]. Under conditions that combine ionizing radiation exposure with ATR/Chk1 dysfunction, the elevated basal level of replication stress in cancer cells may exceed their capacity to maintain genome integrity, leading to the accumulation of unrepaired DNA damage that triggers apoptosis. Consistent with this model, PDAC cells were radiosensitized by Claspin depletion or by Rac1 inhibition, unlike the normal HPNE cells (Figure 6) [23]. The ATR/Chk1 cascade is designed to respond to stalled replication forks, protecting cells from the effects of replication stress, such as that induced by gemcitabine. Rac1 inhibitors should therefore be expected to synergize with gemcitabine and other drugs that produce replication stress. While this therapeutic rationale is promising, further studies are needed to evaluate the clinical utility, selectivity, and safety of Rac1 inhibitors. Although several Rac1 inhibitors have been developed by us and others [9,10,11,12,13,14,15,81], none have yet demonstrated the pharmacokinetic properties required for human trials. To overcome this limitation, our lab and others are now pursuing a new generation of Rac1-targeting compounds using PROTAC technology [82]. These novel agents will trigger Rac1 degradation, providing a potent strategy to suppress ATR/Chk1 signaling and improve tumor sensitivity to standard therapies like gemcitabine.

## 5. Conclusions

These findings identify Rac1 as a critical regulator of ATR/Chk1 signaling through stabilization of the fork protection protein Claspin. Rac1 inhibition promotes the βTrCP-dependent, proteasome-mediated degradation of Claspin via its phosphodegron, thereby impairing Chk1 activation in response to DNA damage. Although Rac1 inhibition reduces Claspin levels in both normal and pancreatic cancer cells, PDAC cells are selectively sensitized to genotoxic stress due to their heightened replication stress and dependence on ATR/Chk1 signaling for survival. Thus, targeting Rac1 represents a promising therapeutic strategy to exploit replication stress and overcome radioresistance in PDAC.

## Data Availability

The original contributions presented in this study are included in the article/Supplementary Materials. Further inquiries can be directed to the corresponding author.

## Supplementary Materials

The following supporting information can be downloaded at: https://www.mdpi.com/article/10.3390/cancers18121908/s1, Figure S1: PLK1 inhibition does not prevent Claspin degradation induced by NSC23766; scans of all individual blots used for generating the Western blot figures.
