# Supplementary material for: Rac1 GTPase Regulates the SCFβTrCP-Mediated Degradation of Claspin and the Cellular Response of Pancreatic Cancer Cells to Gamma Rays
Source: Cancers (Basel). 2026 Jun 11;18(12):1908. doi: 10.3390/cancers18121908 (PMC13296905; doi:10.3390/cancers18121908)
Supplement: Supplementary file 1 [file cancers-18-01908-s001.zip › cancers-4345252-supplementary.pdf]

Supplemental Materials

Figure Legend

Fig. S1. PLK1 inhibition does not prevent Claspin degradation induced by NSC23766. HPAF/CD18 cells were first exposed to PLK1 inhibitor Volasertib at 500 nM [1] or vehicle (DMSO). After 2 hours, NSC23766 (100 μM) was added and samples were collected at the indicated times to monitor Claspin levels. GAPDH was used as an invariant internal control. The experiment was repeated once with the same outcomes.

References

[1] D. Rudolph, M. Steegmaier, M. Hoffmann, M. Grauert, A. Baum, J. Quant, C. Haslinger, P. Garin-Chesa, G.R. Adolf, BI 6727, a Polo-like kinase inhibitor with improved pharmacokinetic profile and broad antitumor activity, Clin Cancer Res, 15 (2009) 3094-3102.

Figure S1

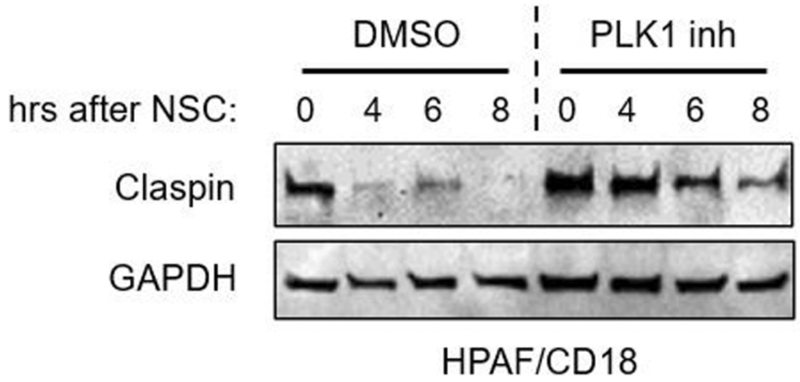
DMSO
PLK1 inh
hrs after NSC:
0
4
6
8
0
4
6
8
Claspin
GAPDH
HPAF/CD18

Figure 1A – Set 1

Set 1 - gel 2

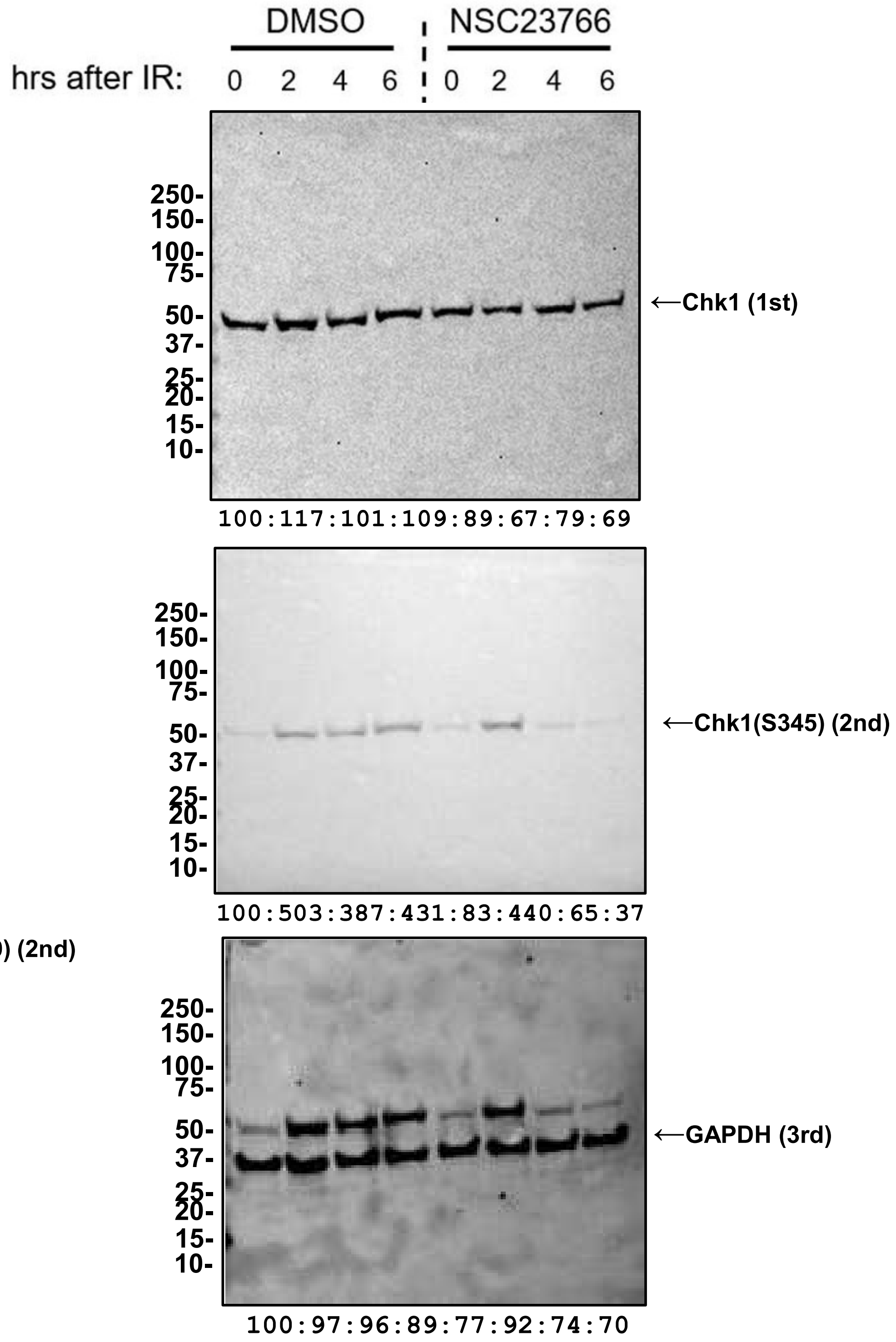

Set 1 - gel 1

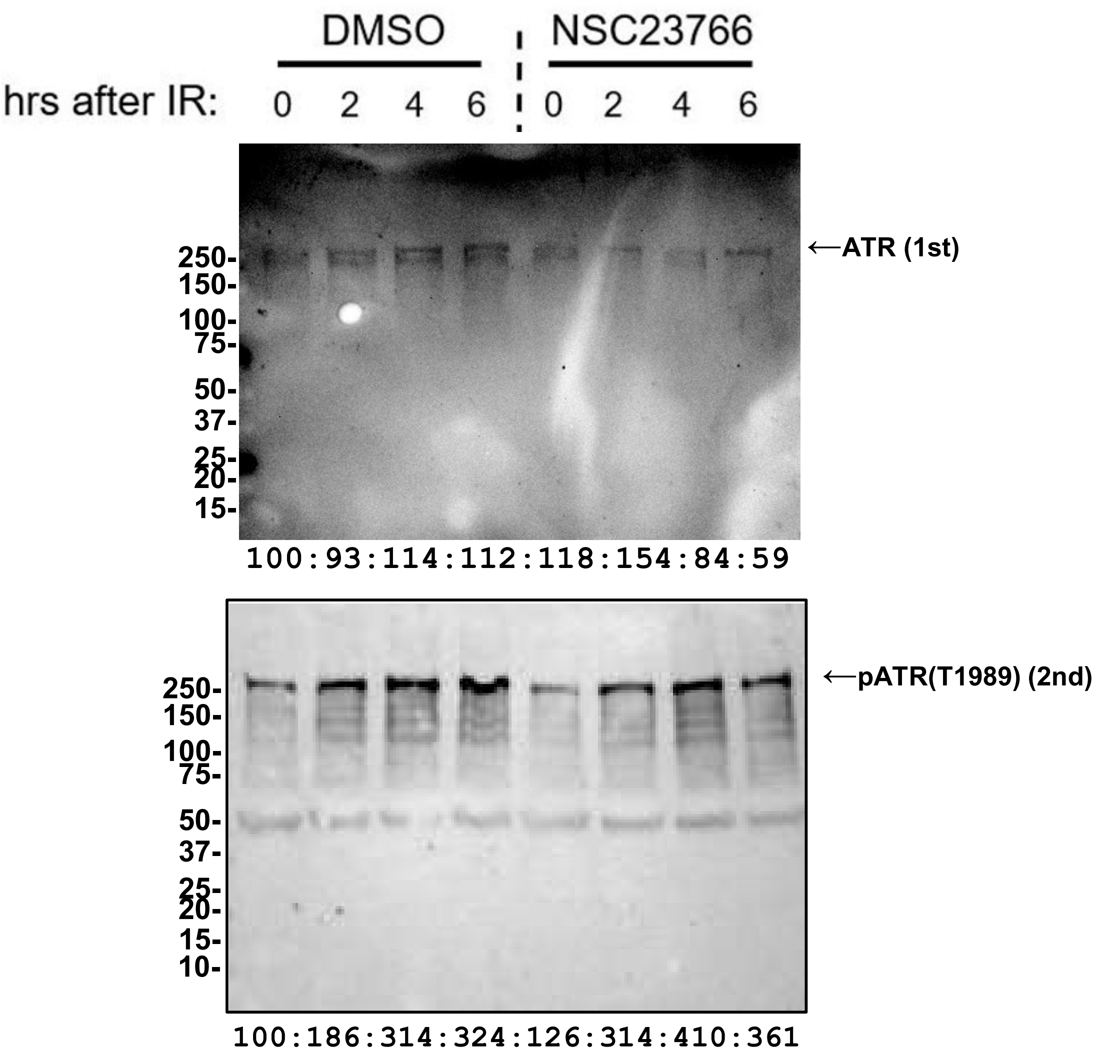

Figure 1A – Set 2

Set 2 - gel 1

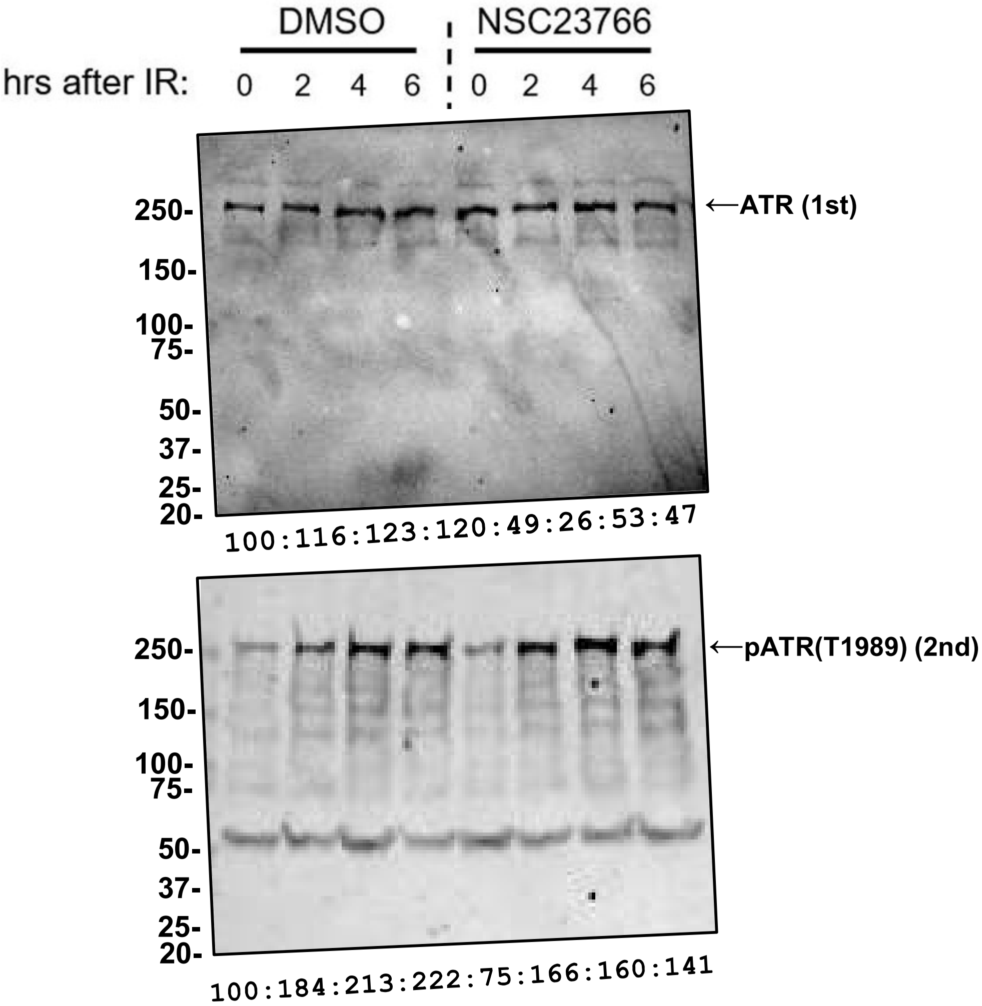

Set 2 - gel 2

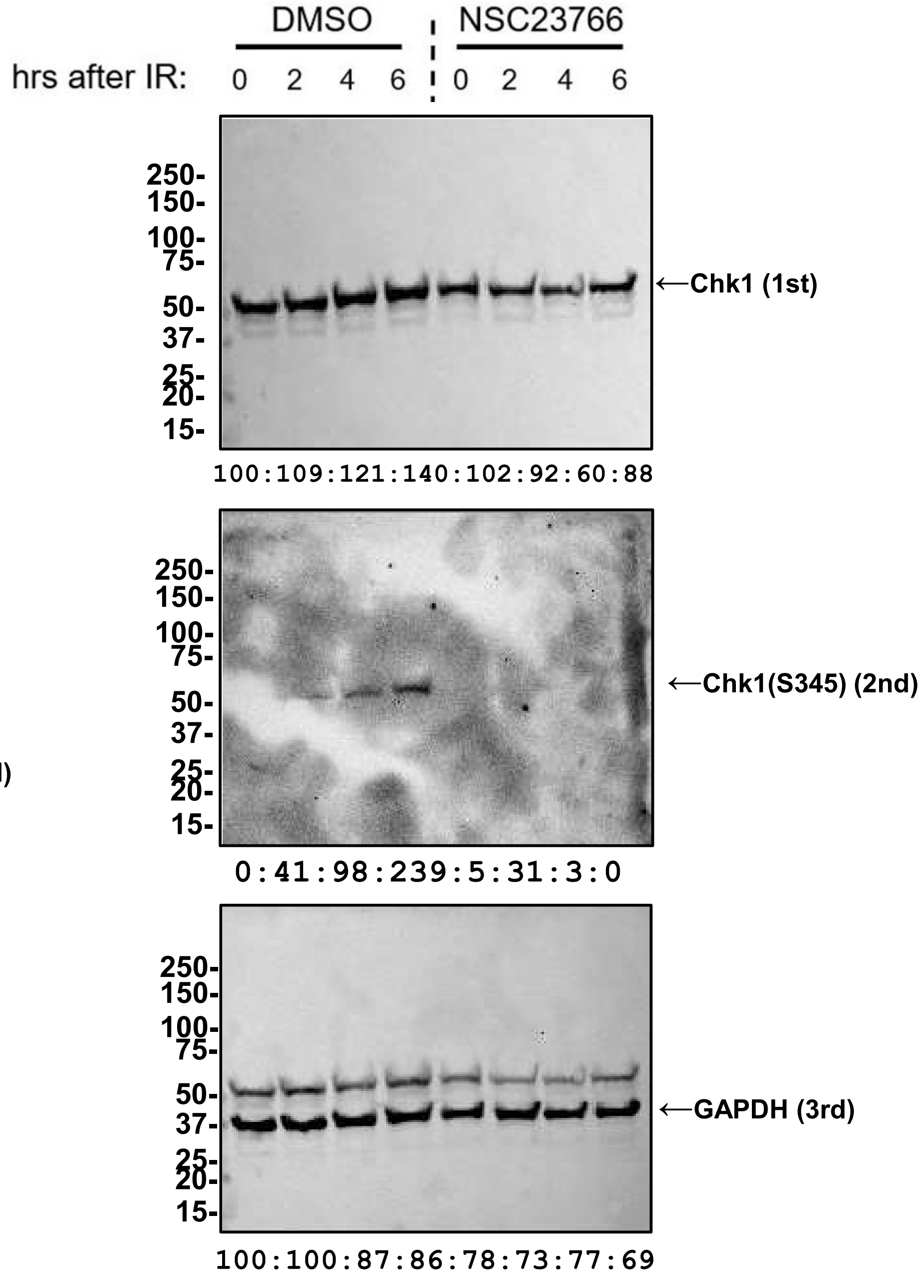

Figure 1B

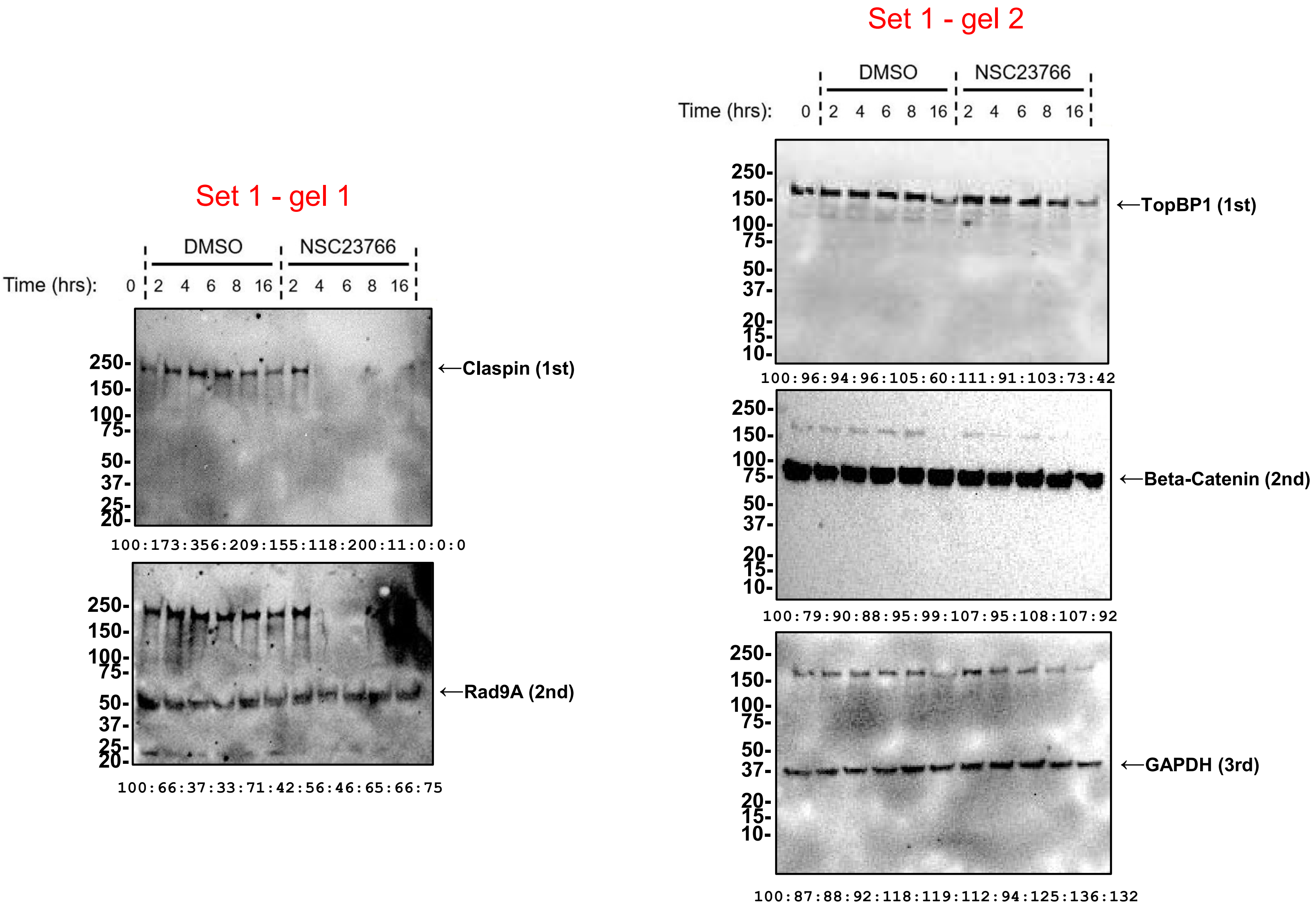
Set 1 - gel 1
DMSO
NSC23766
Time (hrs): 0 2 4 6 8 16 2 4 6 8 16
250-
150-
100-
75-
50-
37-
25-
20-
←Claspin (1st)
100:173:356:209:155:118:200:11:0:0:0
←Rad9A (2nd)
100:66:37:33:71:42:56:46:65:66:75
Set 1 - gel 2
DMSO
NSC23766
Time (hrs): 0 2 4 6 8 16 2 4 6 8 16
250-
150-
100-
75-
50-
37-
20-
15-
10-
←TopBP1 (1st)
100:96:94:96:105:60:111:91:103:73:42
←Beta-Catenin (2nd)
100:79:90:88:95:99:107:95:108:107:92
←GAPDH (3rd)
100:87:88:92:118:119:112:94:125:136:132

Figure 1B

Set 2 – gel1

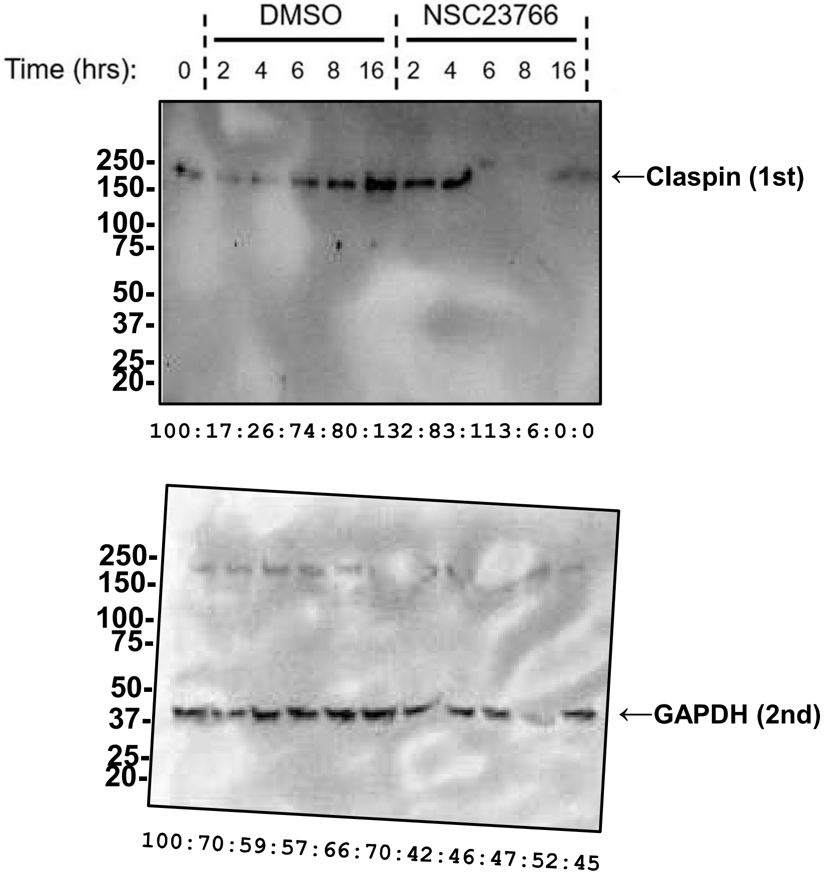

Set 2 – gel2

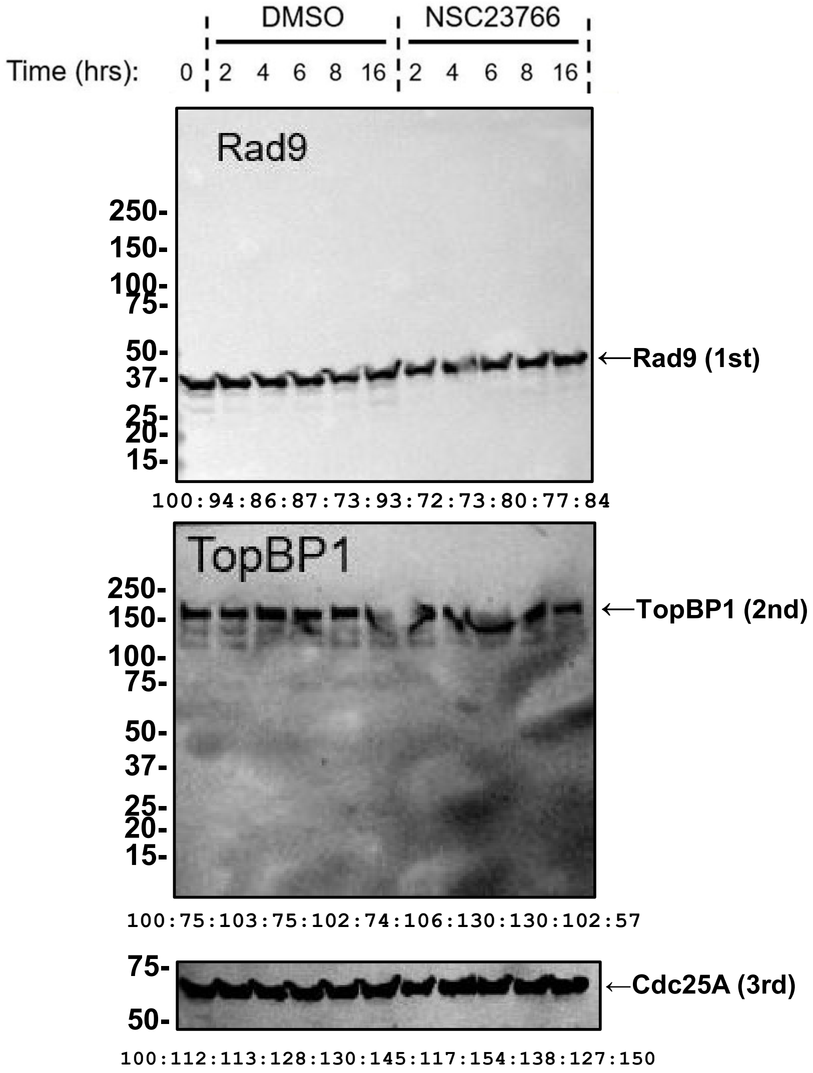

Figure 1C

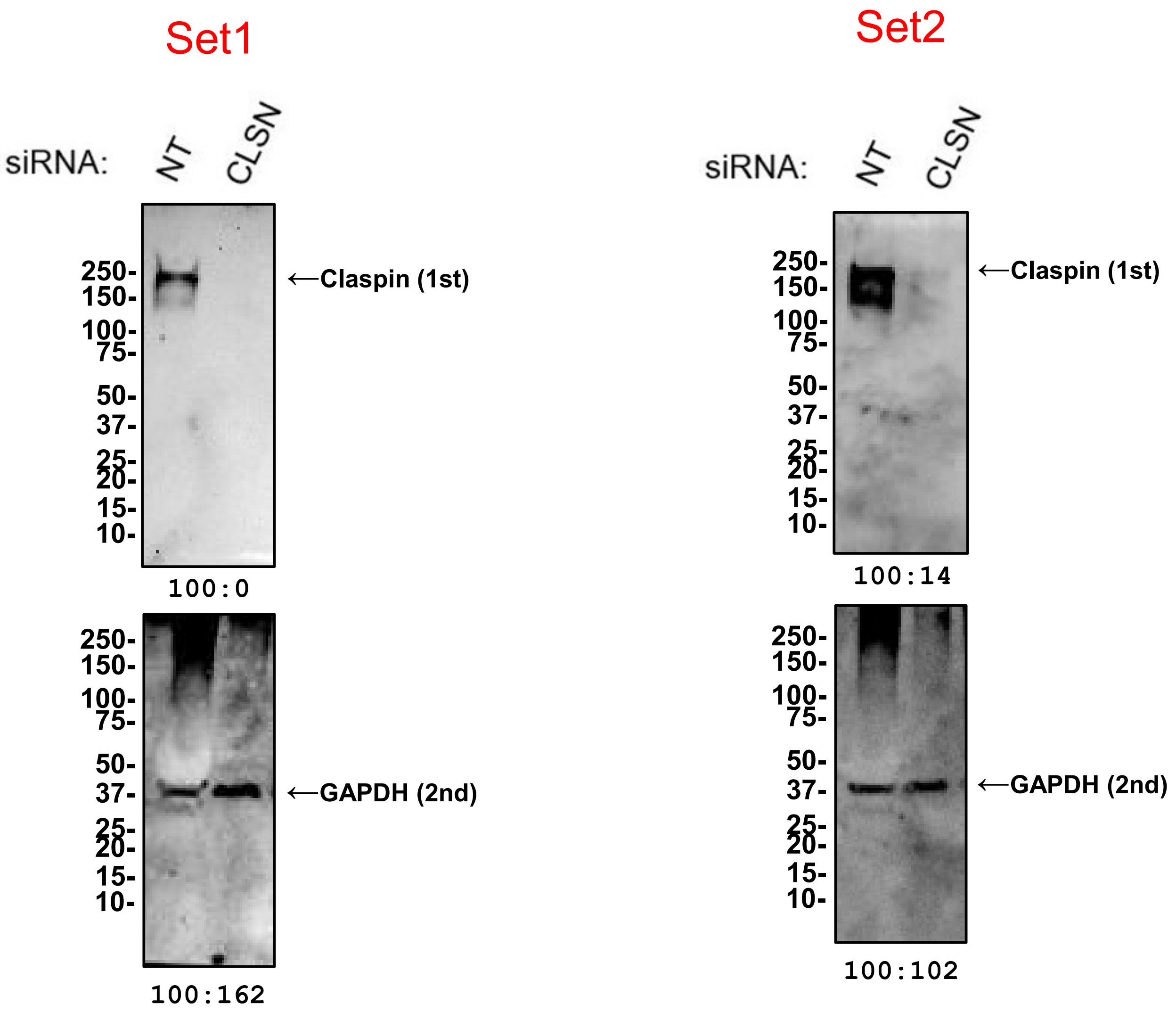
Set1
Set2
siRNA:
NT
CLSN
250-
150-
100-
75-
50-
37-
25-
20-
15-
10-
←Claspin (1st)
100:0
←GAPDH (2nd)
100:162
100:14
100:102

Figure 1C

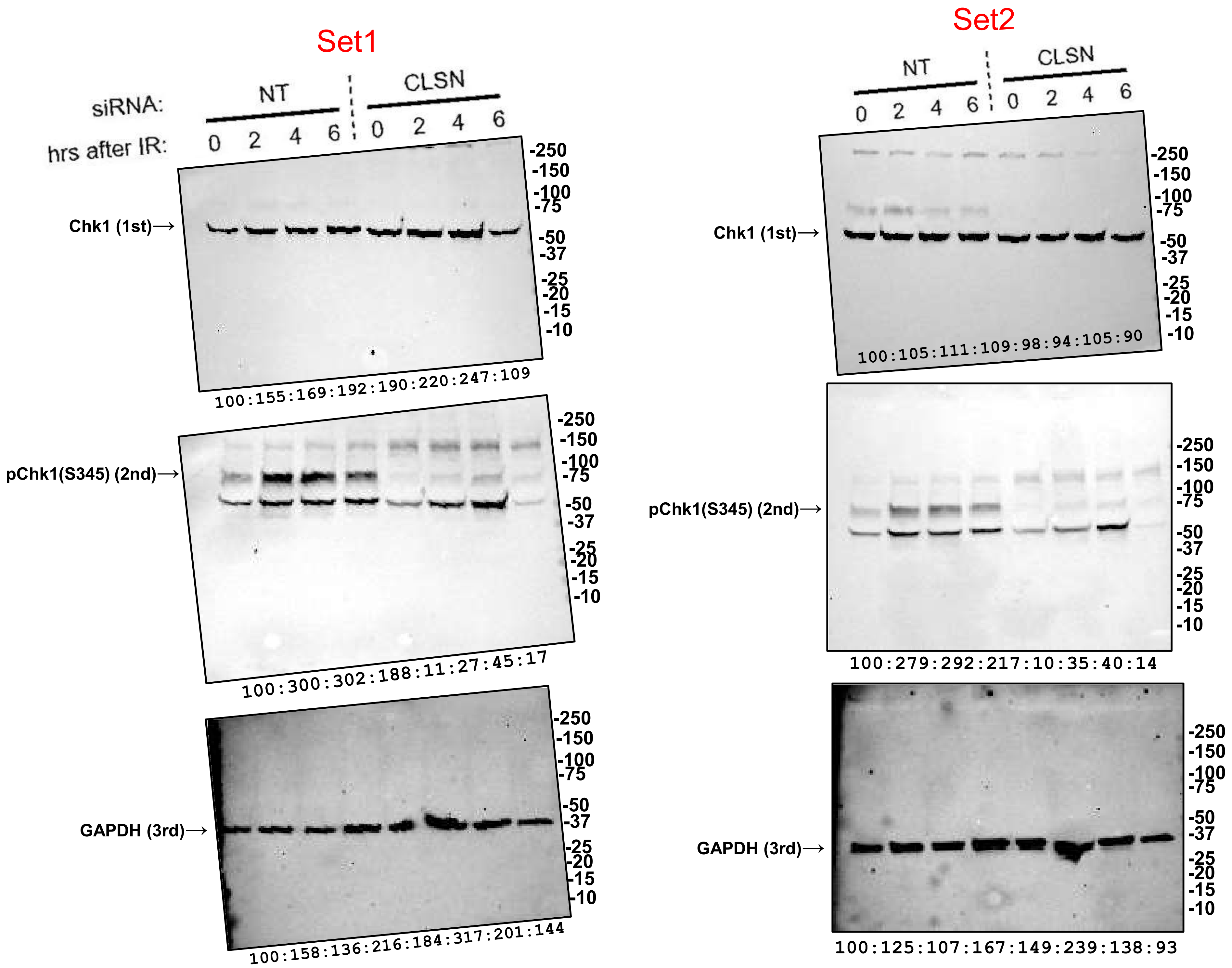

Set1
siRNA:
NT
CLSN
hrs after IR:
0 2 4 6 0 2 4 6
Chk1 (1st)→
-250 -150 -100 -75 -50 -37 -25 -20 -15 -10
100:155:169:192:190:220:247:109
pChk1(S345) (2nd)→
100:300:302:188:11:27:45:17
GAPDH (3rd)→
100:158:136:216:184:317:201:144
Set2
NT
CLSN
0 2 4 6 0 2 4 6
Chk1 (1st)→
100:105:111:109:98:94:105:90
pChk1(S345) (2nd)→
100:279:292:217:10:35:40:14
GAPDH (3rd)→
100:125:107:167:149:239:138:93

Figure 2A

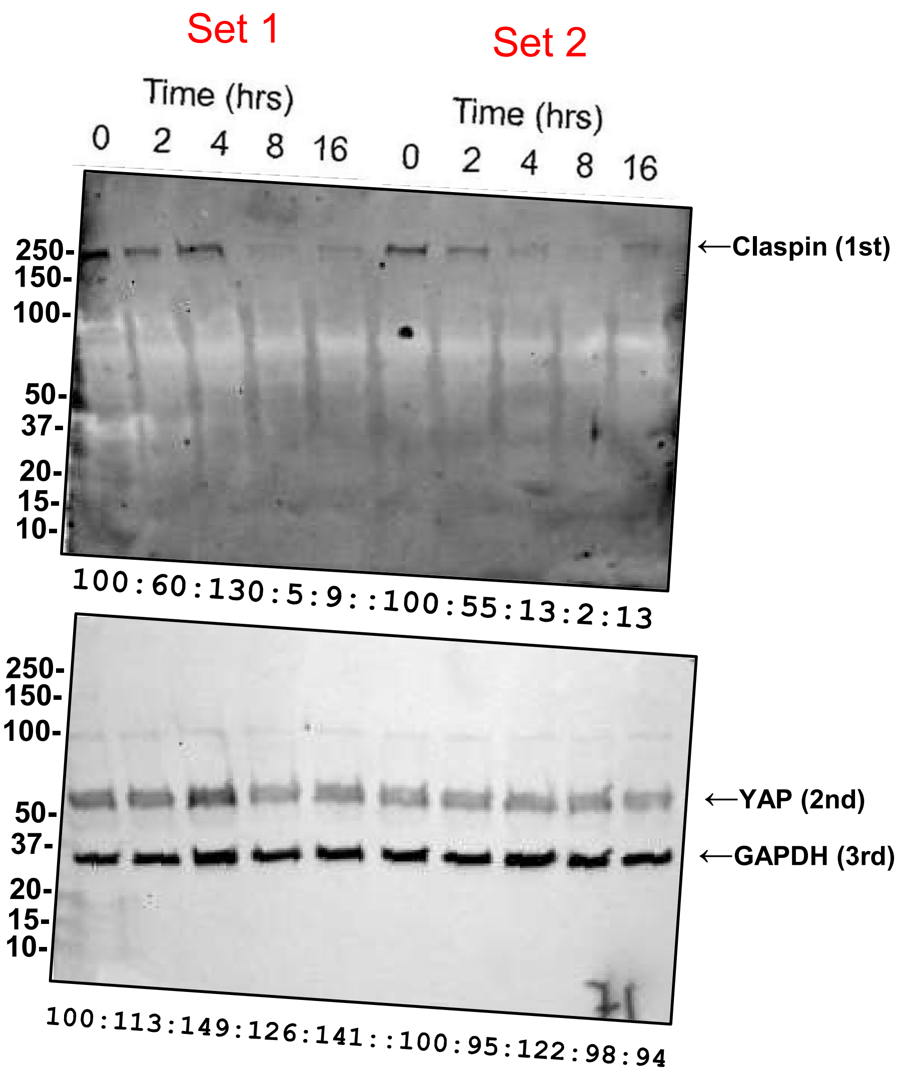

Set 1
Set 2
Time (hrs)
0 2 4 8 16
Time (hrs)
0 2 4 8 16
250-
150-
100-
50-
37-
20-
15-
10-
←Claspin (1st)
100:60:130:5:9::100:55:13:2:13
250-
150-
100-
50-
37-
20-
15-
10-
←YAP (2nd)
←GAPDH (3rd)
100:113:149:126:141::100:95:122:98:94

Figure 2B

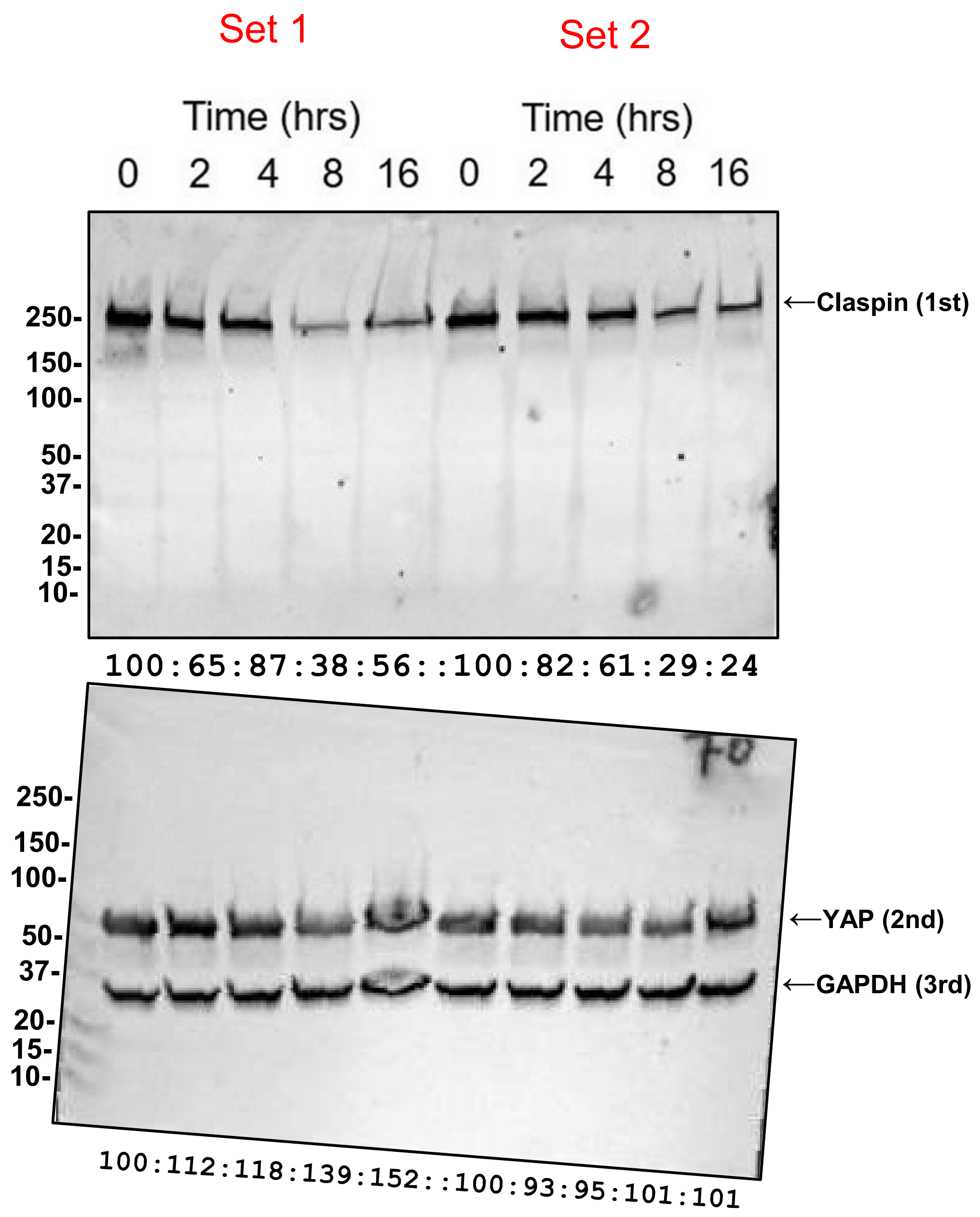

Set 1
Set 2
Time (hrs)
Time (hrs)
0 2 4 8 16 0 2 4 8 16
250-
150-
100-
50-
37-
20-
15-
10-
←Claspin (1st)
100:65:87:38:56::100:82:61:29:24
70
250-
150-
100-
50-
37-
20-
15-
10-
←YAP (2nd)
←GAPDH (3rd)
100:112:118:139:152::100:93:95:101:101

Figure 2C

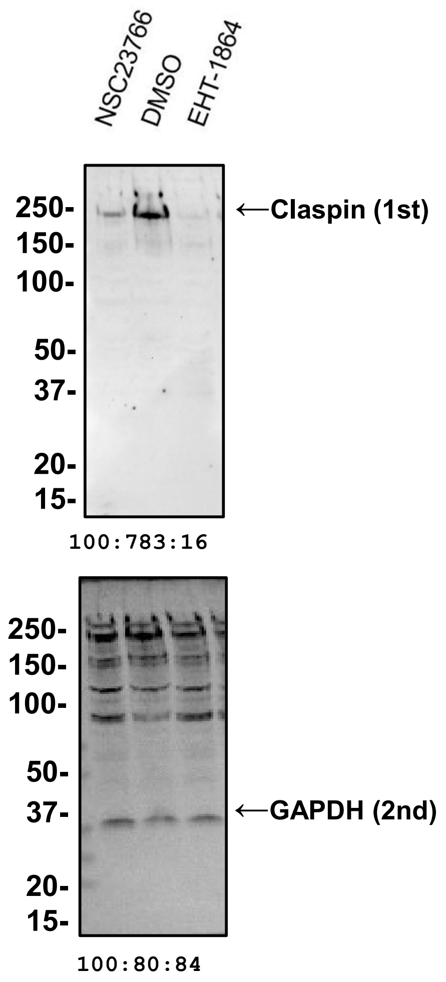
NSC23766
DMSO
EHT-1864
250-
150-
100-
50-
37-
20-
15-
←Claspin (1st)
100:783:16
250-
150-
100-
50-
37-
20-
15-
←GAPDH (2nd)
100:80:84

Figure 2D

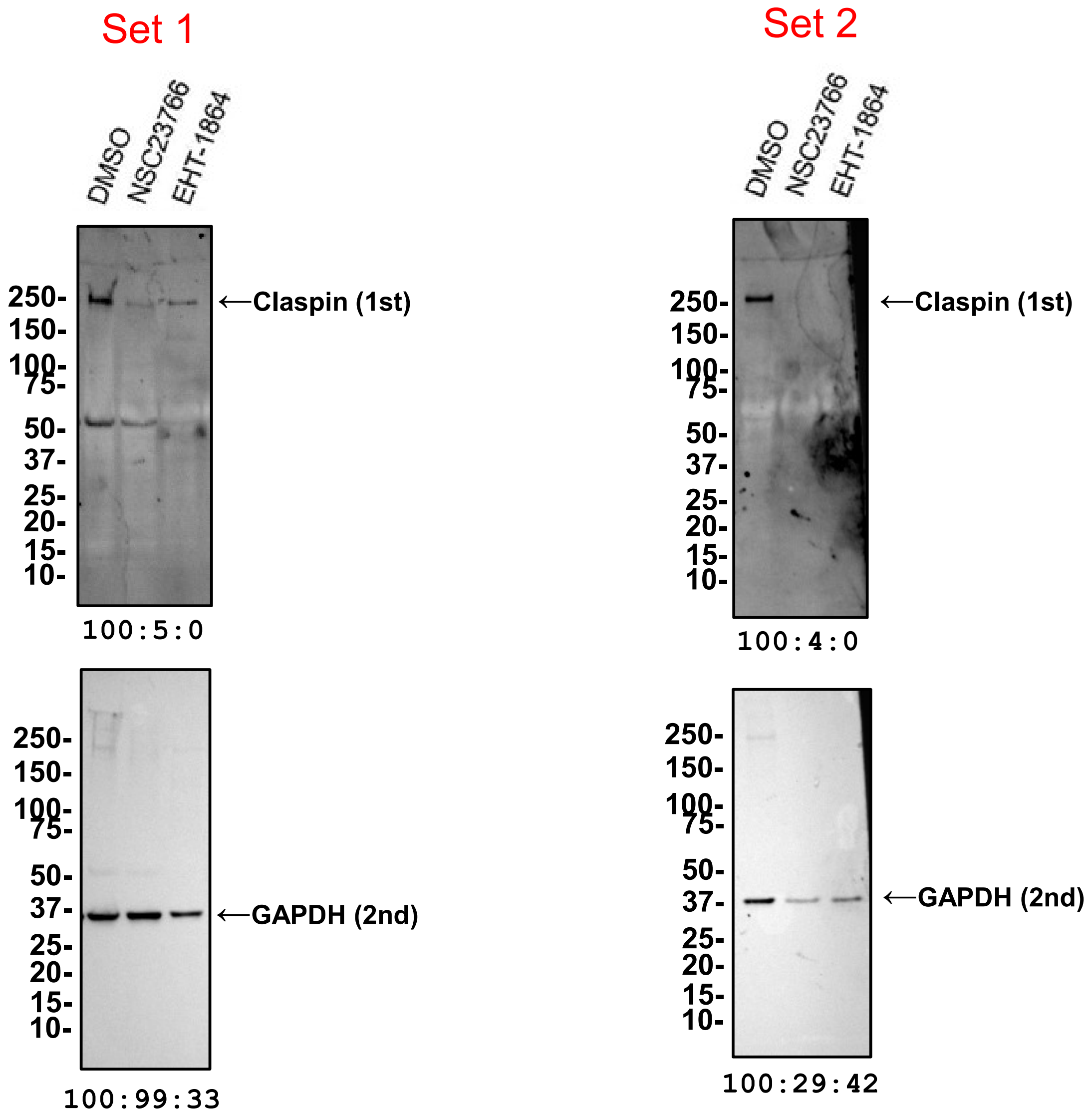
Set 1
Set 2
DMSO
NSC23766
EHT-1864
250-
150-
100-
75-
50-
37-
25-
20-
15-
10-
Claspin (1st)
GAPDH (2nd)
100:5:0
100:99:33
100:4:0
100:29:42

Figure 2E

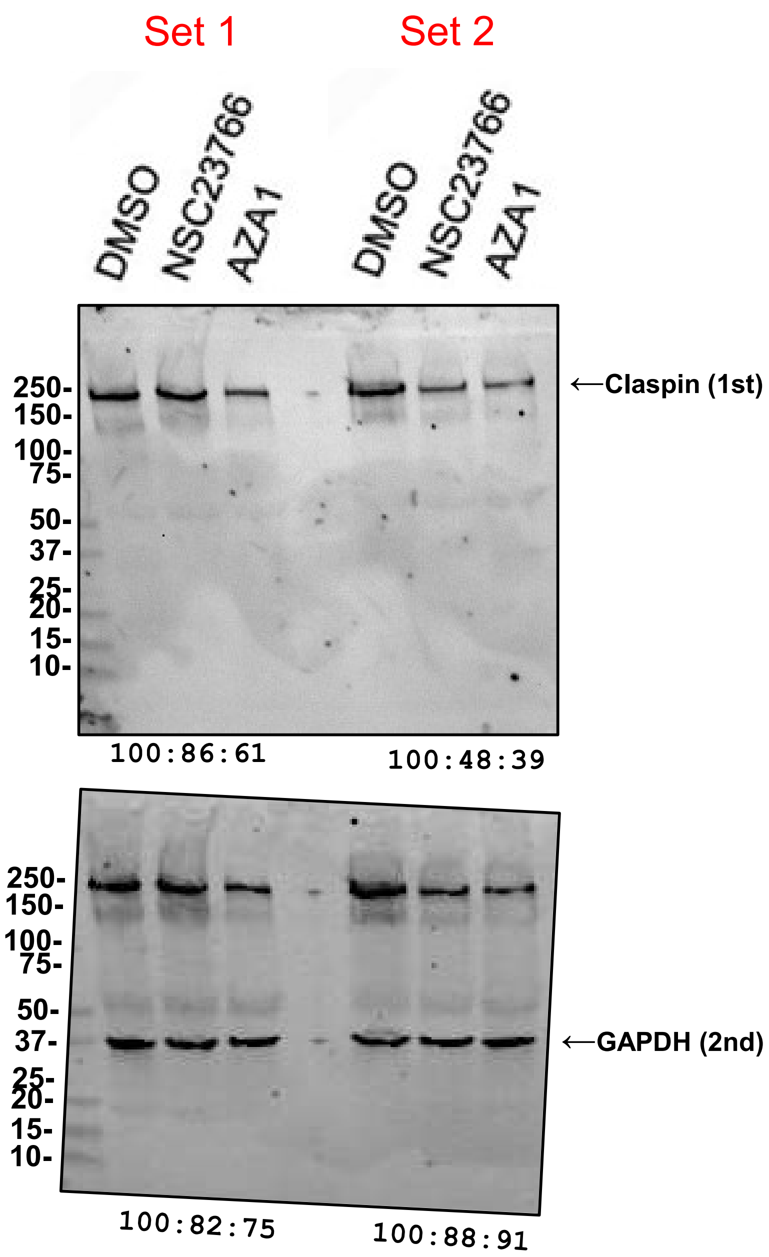

Set 1
Set 2
DMSO
NSC23766
AZA1
DMSO
NSC23766
AZA1
250-
150-
100-
75-
50-
37-
25-
20-
15-
10-
←Claspin (1st)
100:86:61
100:48:39
250-
150-
100-
75-
50-
37-
25-
20-
15-
10-
←GAPDH (2nd)
100:82:75
100:88:91

Figure 3A

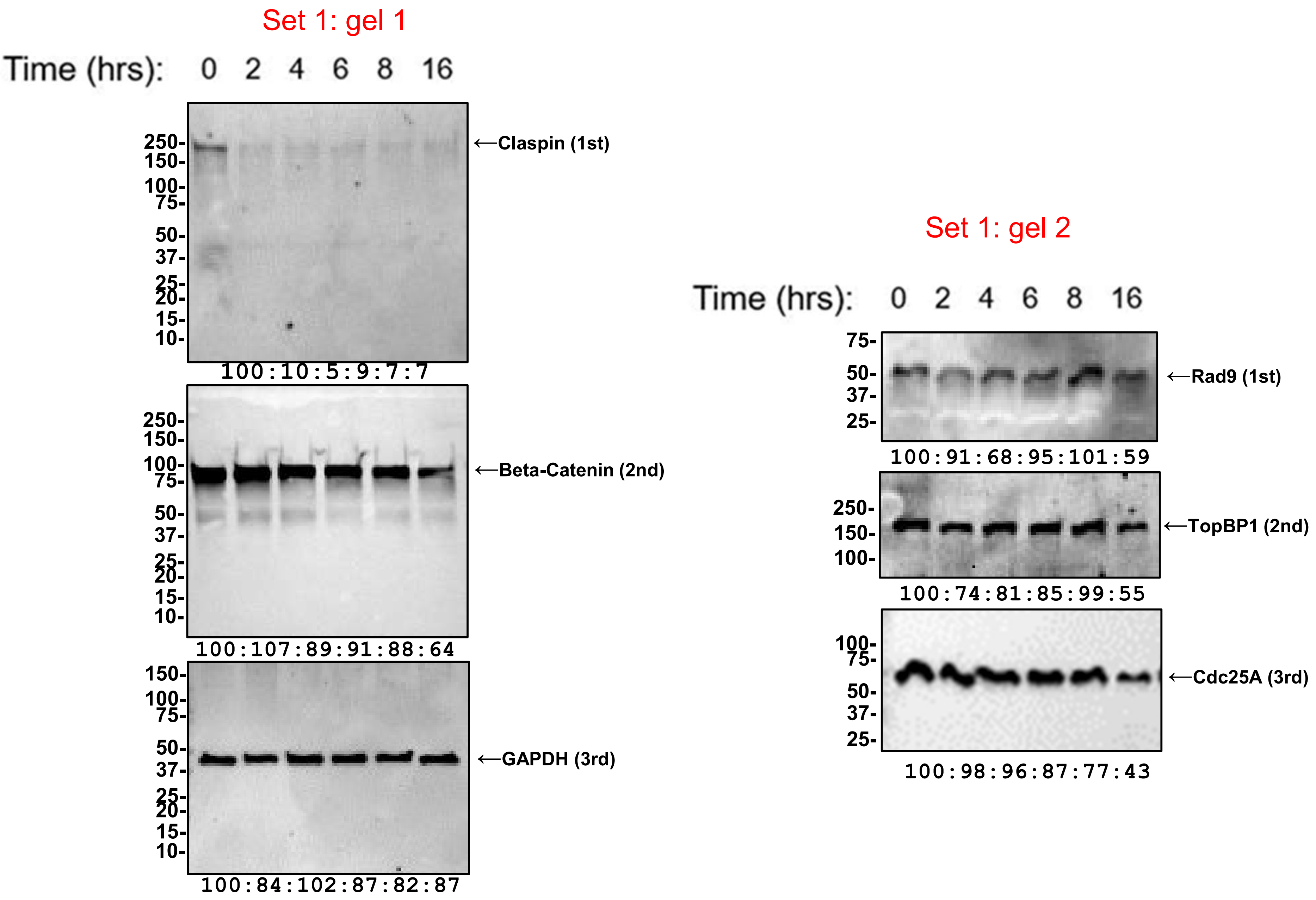

Set 1: gel 1
Time (hrs): 0 2 4 6 8 16
250-
150-
100-
75-
50-
37-
25-
20-
15-
10-
←Claspin (1st)
100:10:5:9:7:7
←Beta-Catenin (2nd)
100:107:89:91:88:64
←GAPDH (3rd)
100:84:102:87:82:87
Set 1: gel 2
Time (hrs): 0 2 4 6 8 16
←Rad9 (1st)
100:91:68:95:101:59
←TopBP1 (2nd)
100:74:81:85:99:55
←Cdc25A (3rd)
100:98:96:87:77:43

Figure 3A

Set 2: gel1

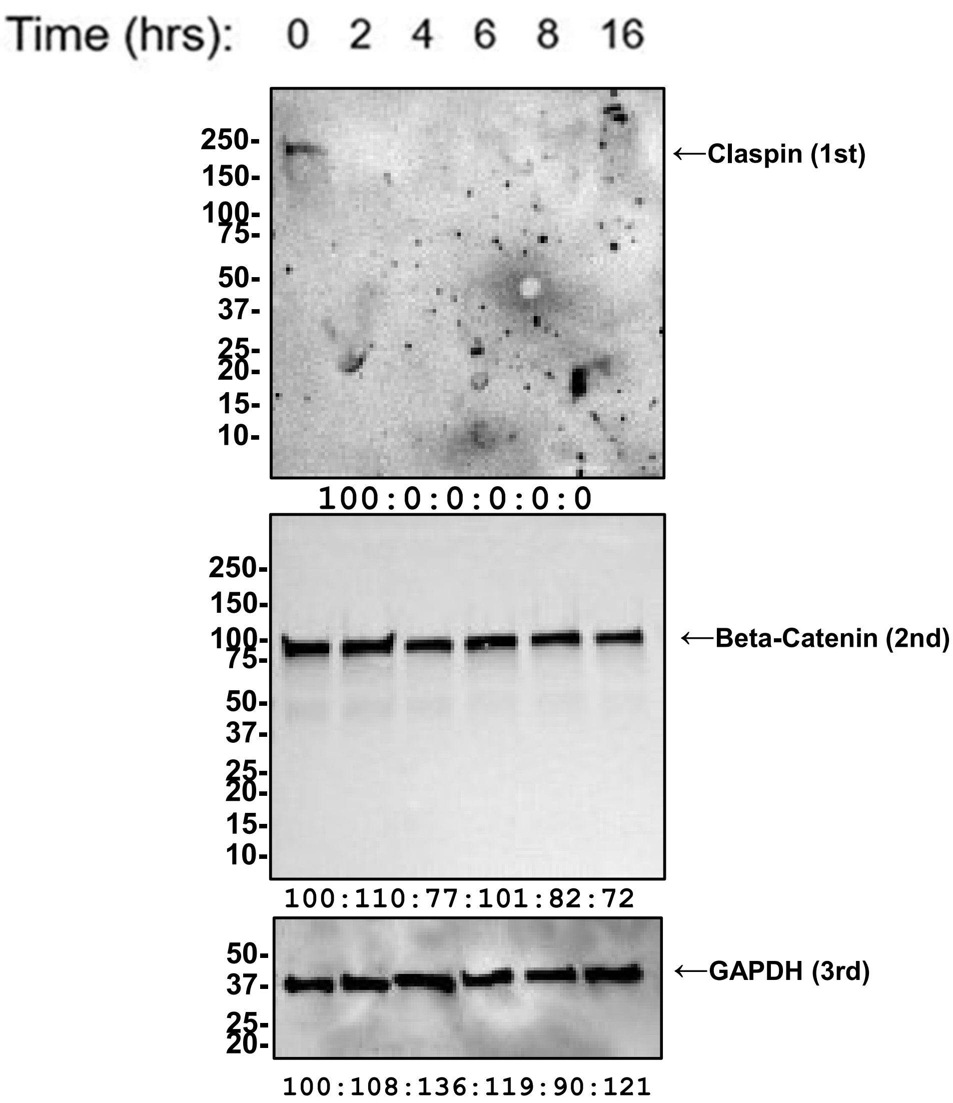

Set 2: gel 2

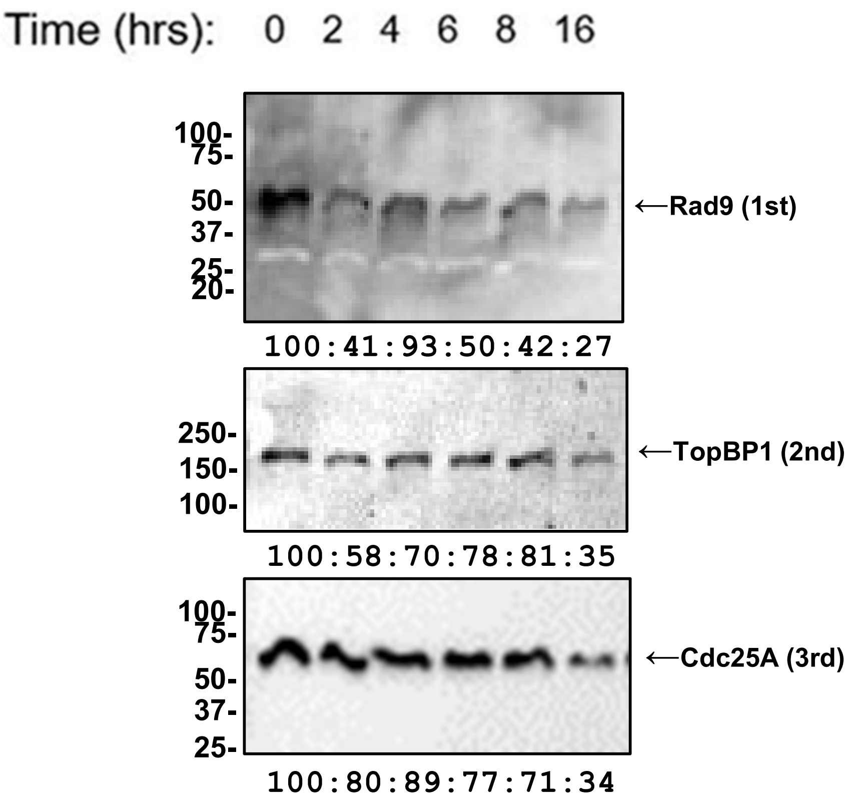

Figure 3B

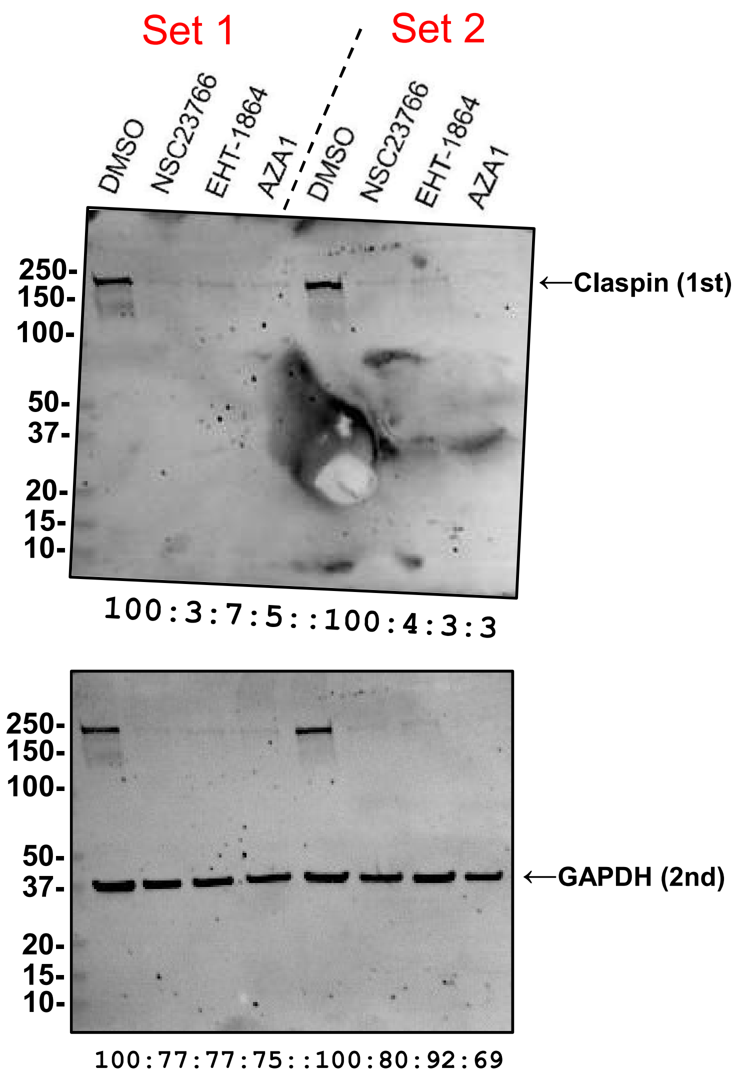

Set 1
Set 2
DMSO
NSC23766
EHT-1864
AZA1
DMSO
NSC23766
EHT-1864
AZA1
250-
150-
100-
50-
37-
20-
15-
10-
←Claspin (1st)
100:3:7:5::100:4:3:3
250-
150-
100-
50-
37-
20-
15-
10-
←GAPDH (2nd)
100:77:77:75::100:80:92:69

Figure 3C

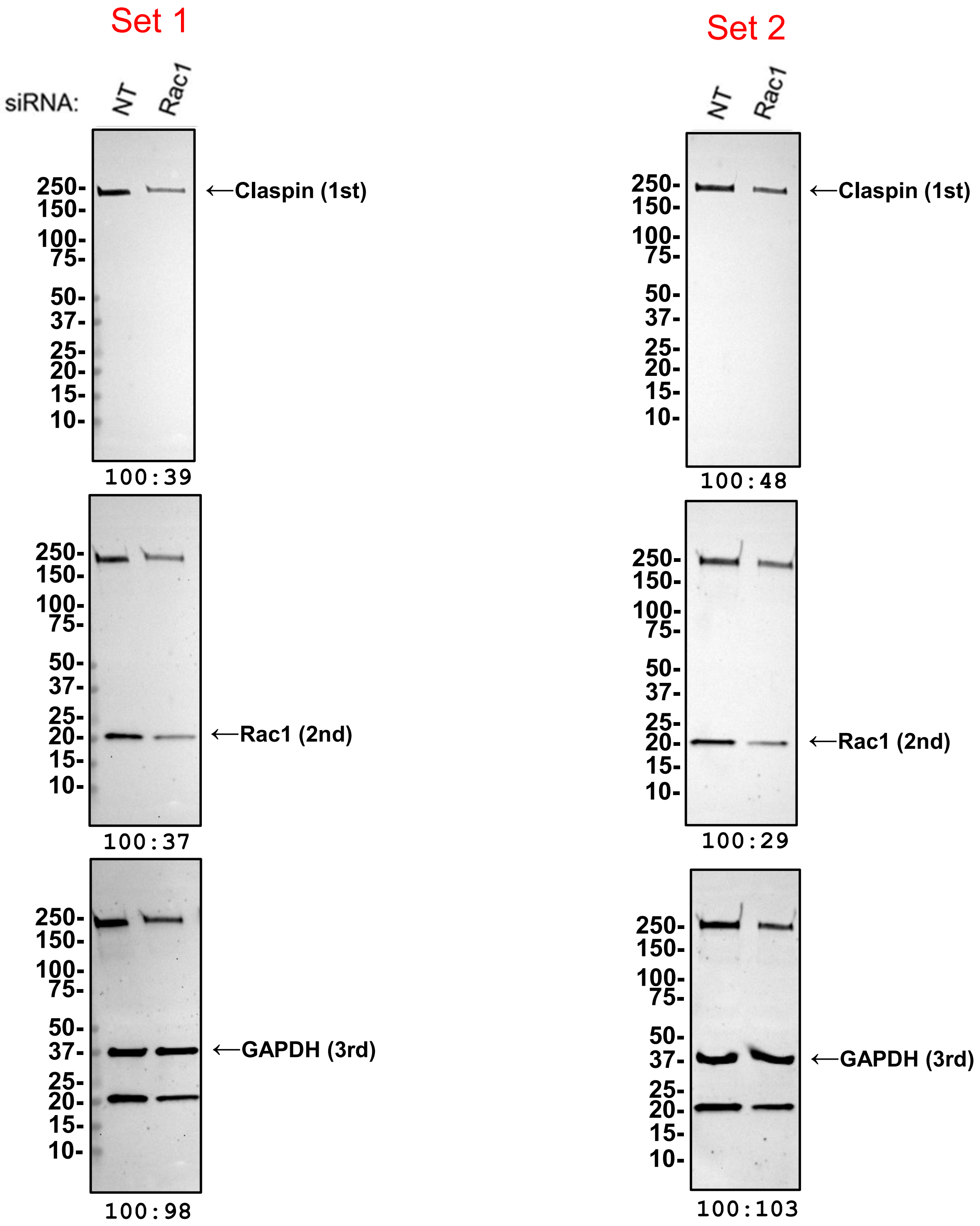

Set 1
Set 2
siRNA:
NT
Rac1
250-
150-
100-
75-
50-
37-
25-
20-
15-
10-
←Claspin (1st)
←Rac1 (2nd)
←GAPDH (3rd)
100:39
100:37
100:98
100:48
100:29
100:103

Figure 4B

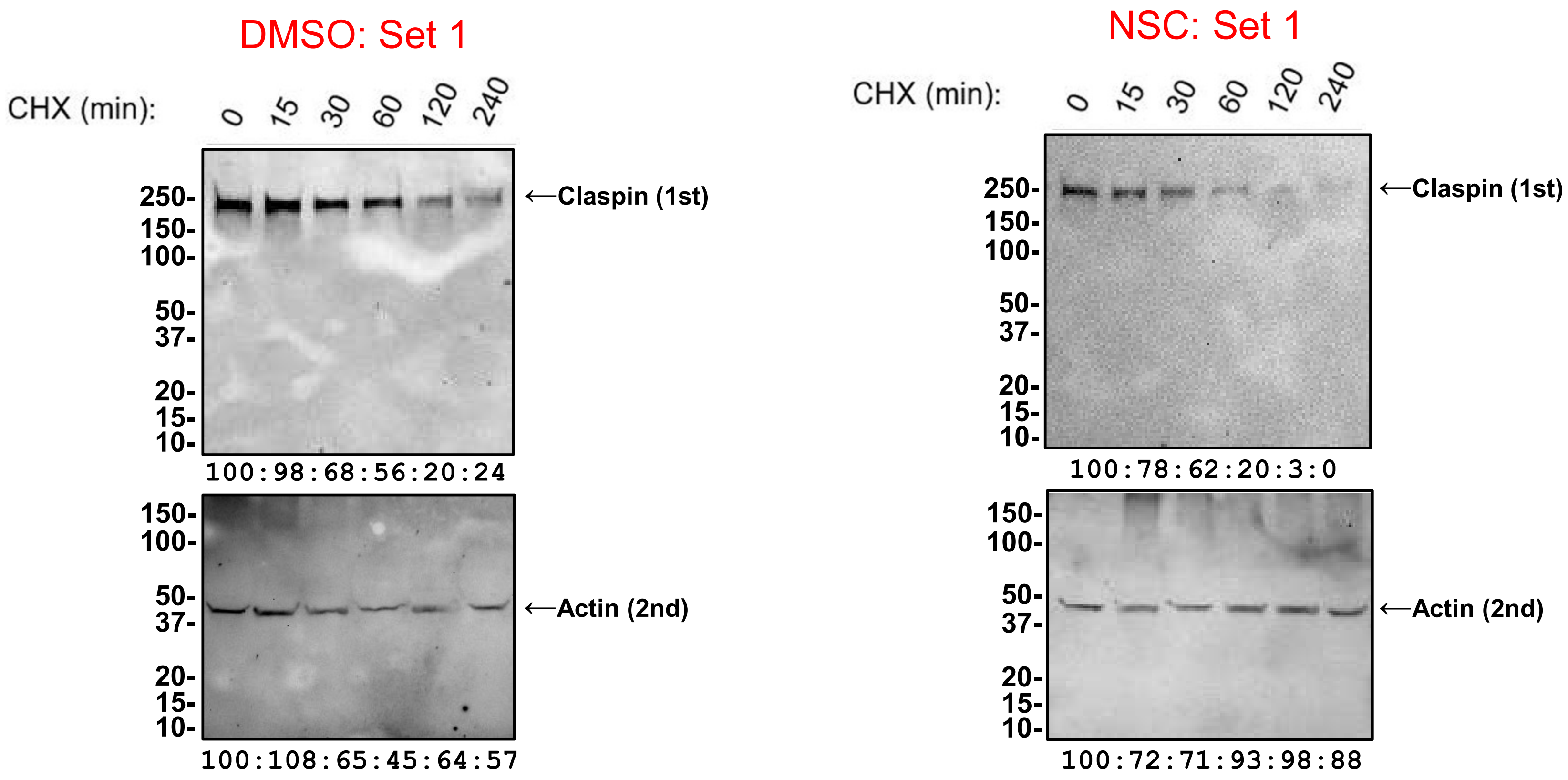
DMSO: Set 1
CHX (min):
0
15
30
60
120
240
250-
150-
100-
50-
37-
20-
15-
10-
←Claspin (1st)
100:98:68:56:20:24
150-
100-
50-
37-
20-
15-
10-
←Actin (2nd)
100:108:65:45:64:57
NSC: Set 1
CHX (min):
0
15
30
60
120
240
250-
150-
100-
50-
37-
20-
15-
10-
←Claspin (1st)
100:78:62:20:3:0
150-
100-
50-
37-
20-
15-
10-
←Actin (2nd)
100:72:71:93:98:88

Figure 4B

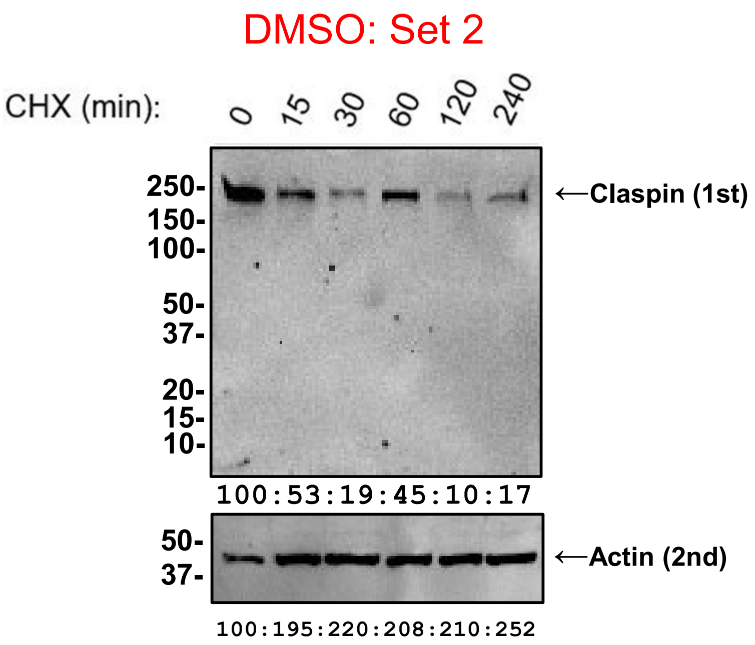

DMSO: Set 2
CHX (min):
0
15
30
60
120
240
250-
150-
100-
50-
37-
20-
15-
10-
←Claspin (1st)
100:53:19:45:10:17
50-
37-
←Actin (2nd)
100:195:220:208:210:252

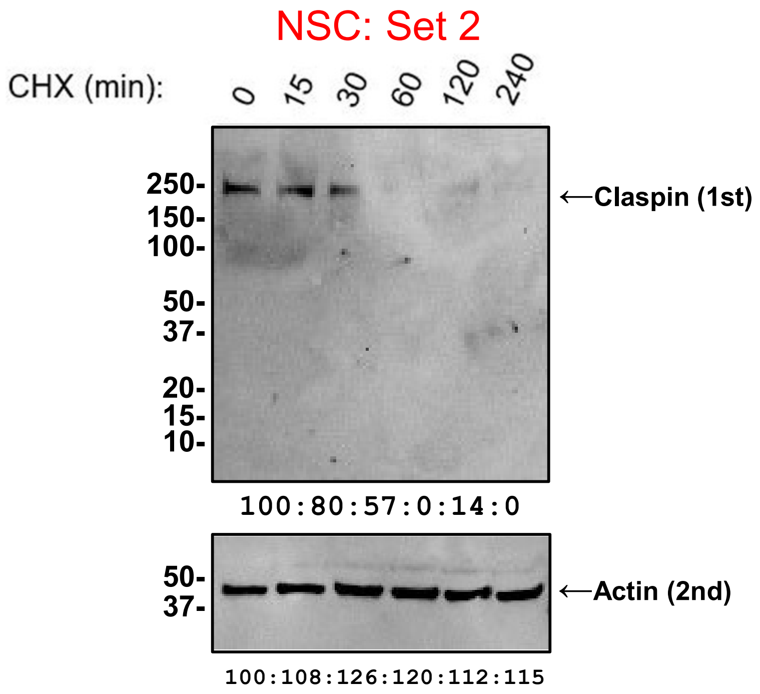

NSC: Set 2
CHX (min):
0
15
30
60
120
240
250-
150-
100-
50-
37-
20-
15-
10-
←Claspin (1st)
100:80:57:0:14:0
50-
37-
←Actin (2nd)
100:108:126:120:112:115

Figure 4C

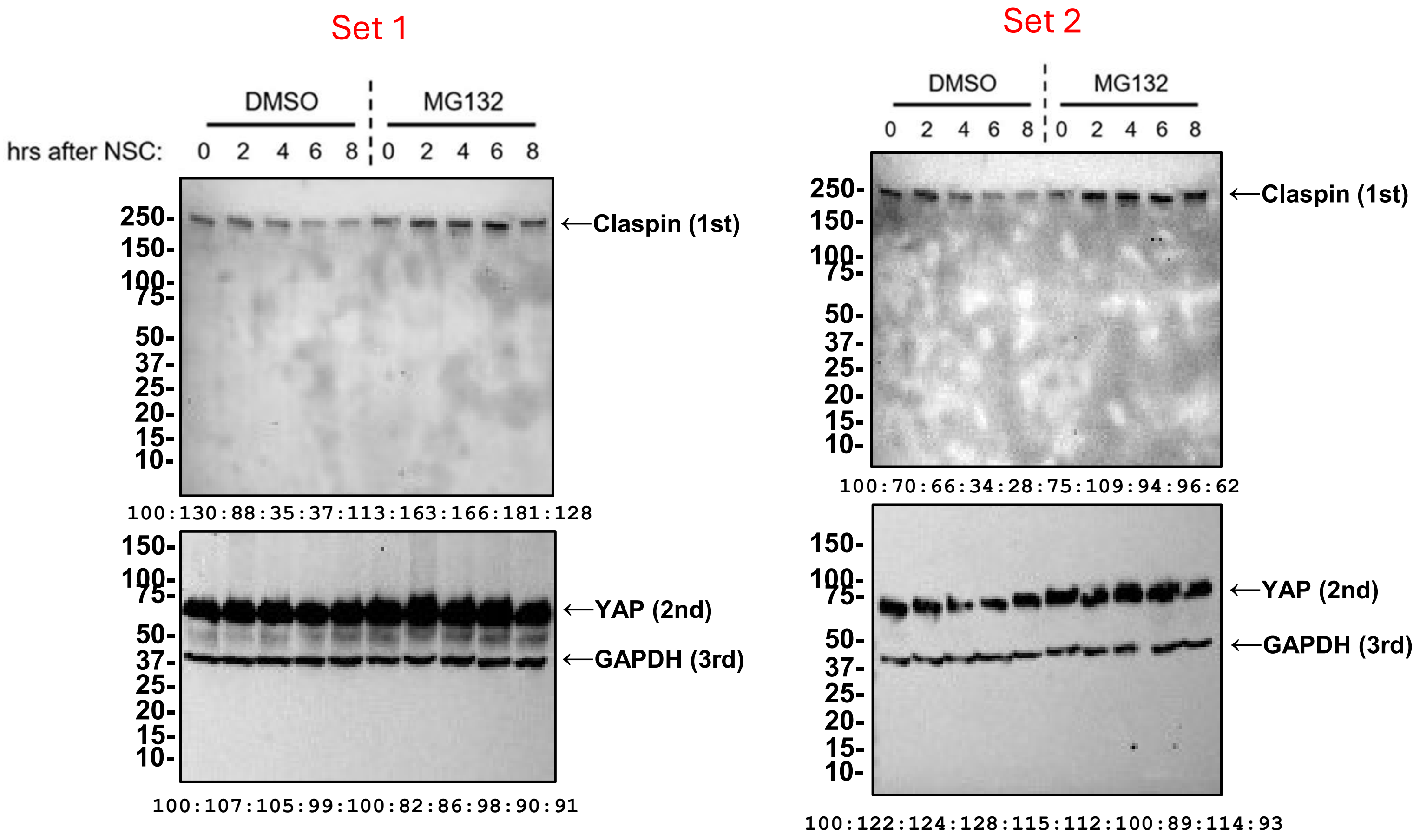
Set 1
DMSO
MG132
hrs after NSC: 0 2 4 6 8 0 2 4 6 8
250-
150-
100-
75-
50-
37-
25-
20-
15-
10-
←Claspin (1st)
100:130:88:35:37:113:163:166:181:128
←YAP (2nd)
←GAPDH (3rd)
100:107:105:99:100:82:86:98:90:91
Set 2
DMSO
MG132
0 2 4 6 8 0 2 4 6 8
←Claspin (1st)
100:70:66:34:28:75:109:94:96:62
←YAP (2nd)
←GAPDH (3rd)
100:122:124:128:115:112:100:89:114:93

Figure 5B

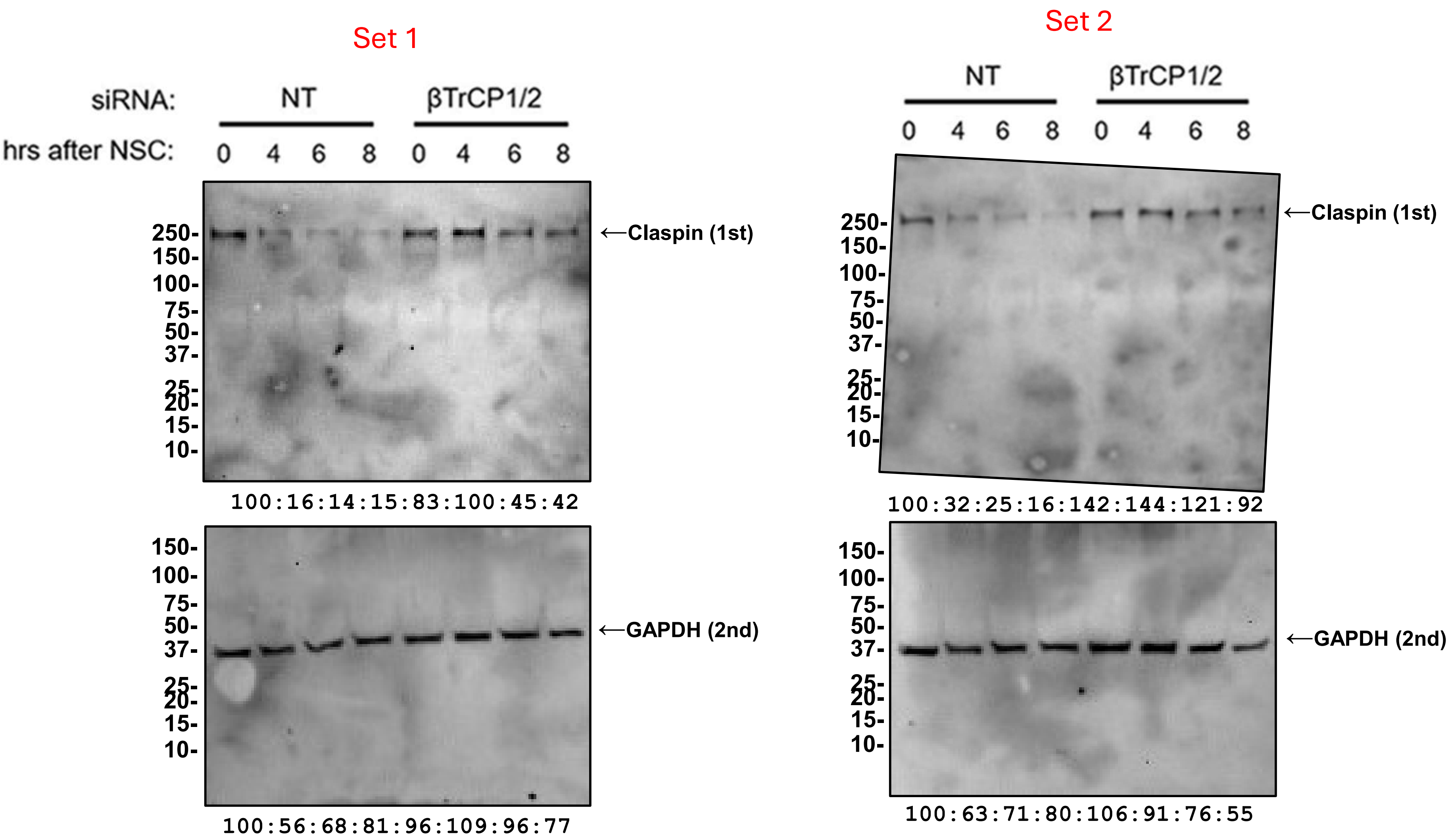
Set 1
Set 2
siRNA:
NT
βTrCP1/2
hrs after NSC:
0 4 6 8 0 4 6 8
250-
150-
100-
75-
50-
37-
25-
20-
15-
10-
←Claspin (1st)
100:16:14:15:83:100:45:42
←GAPDH (2nd)
100:56:68:81:96:109:96:77
100:32:25:16:142:144:121:92
100:63:71:80:106:91:76:55

Figure 5C

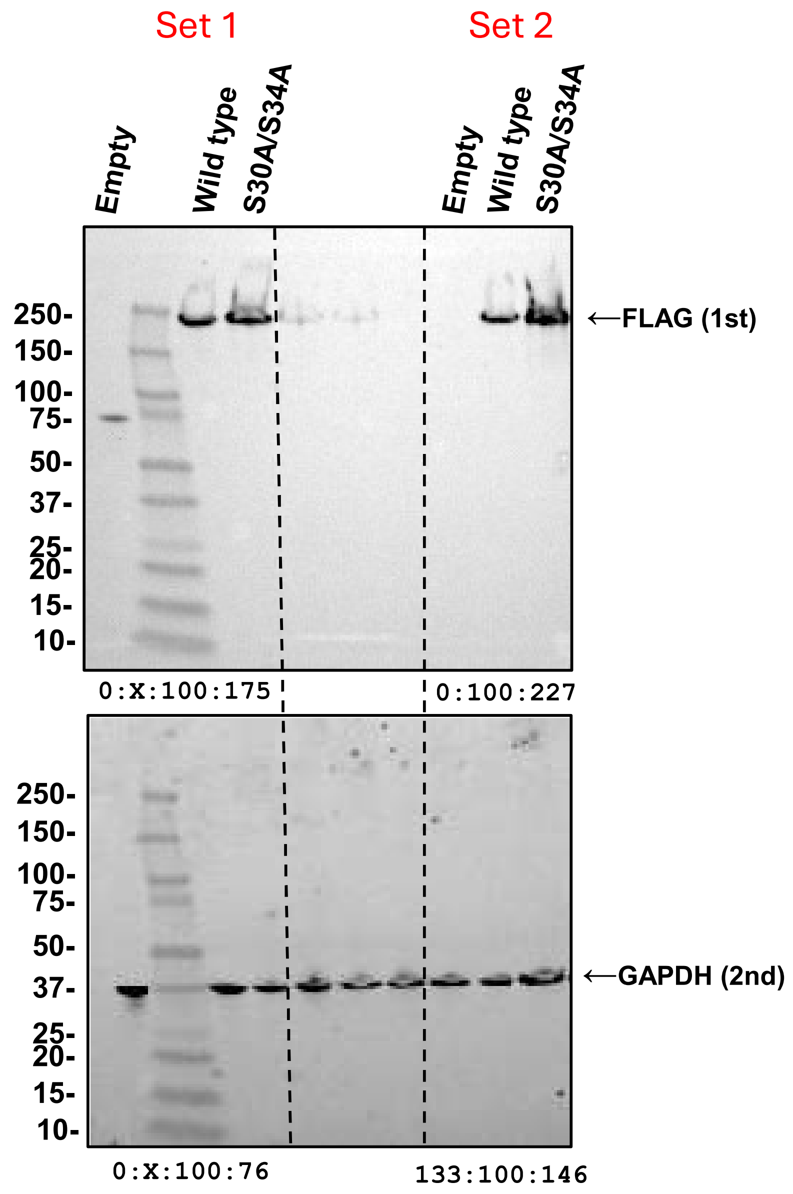

Set 1
Set 2
Empty
Wild type
S30A/S34A
Empty
Wild type
S30A/S34A
250-
150-
100-
75-
50-
37-
25-
20-
15-
10-
←FLAG (1st)
0:X:100:175
0:100:227
250-
150-
100-
75-
50-
37-
25-
20-
15-
10-
←GAPDH (2nd)
0:X:100:76
133:100:146

Figure 5D

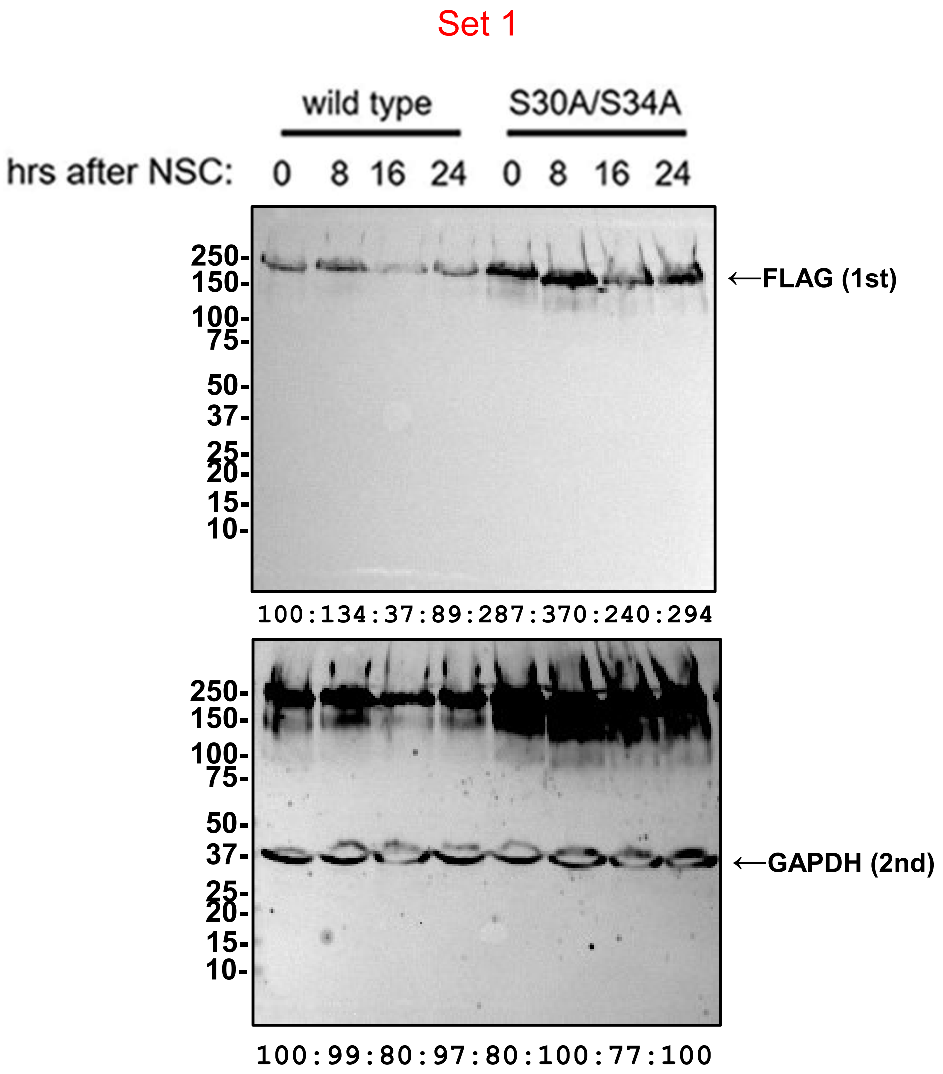
Set 1
wild type
S30A/S34A
hrs after NSC:
0 8 16 24 0 8 16 24
250-
150-
100-
75-
50-
37-
25-
20-
15-
10-
←FLAG (1st)
100:134:37:89:287:370:240:294
←GAPDH (2nd)
100:99:80:97:80:100:77:100

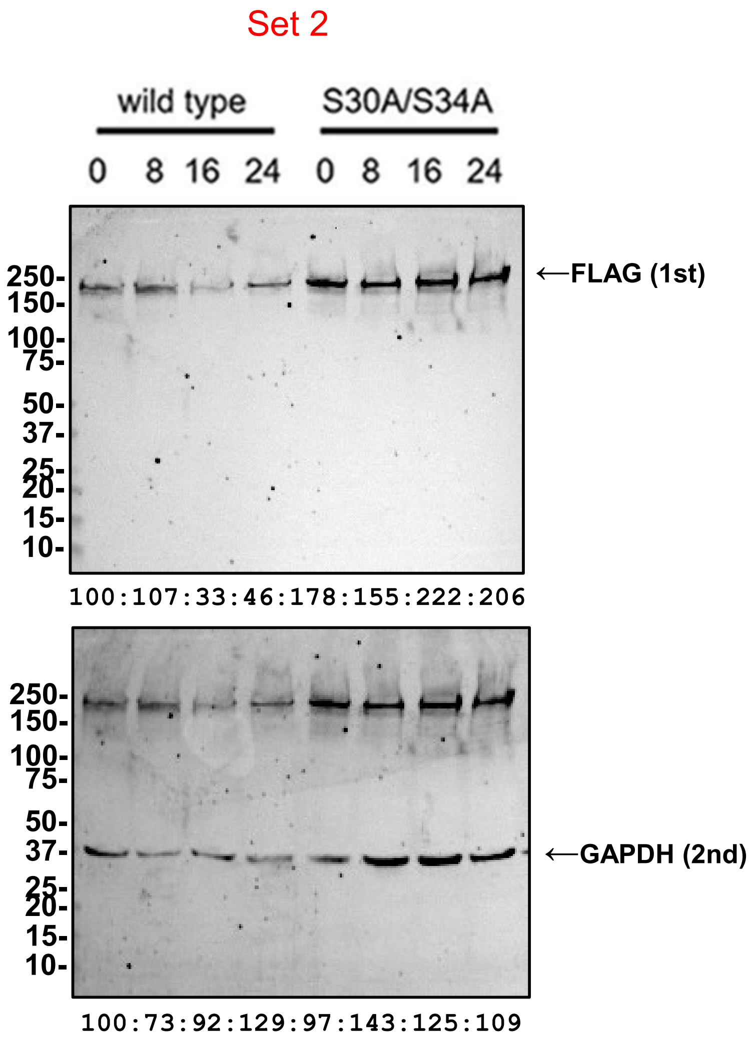
Set 2
wild type
S30A/S34A
0 8 16 24 0 8 16 24
250-
150-
100-
75-
50-
37-
25-
20-
15-
10-
←FLAG (1st)
100:107:33:46:178:155:222:206
←GAPDH (2nd)
100:73:92:129:97:143:125:109

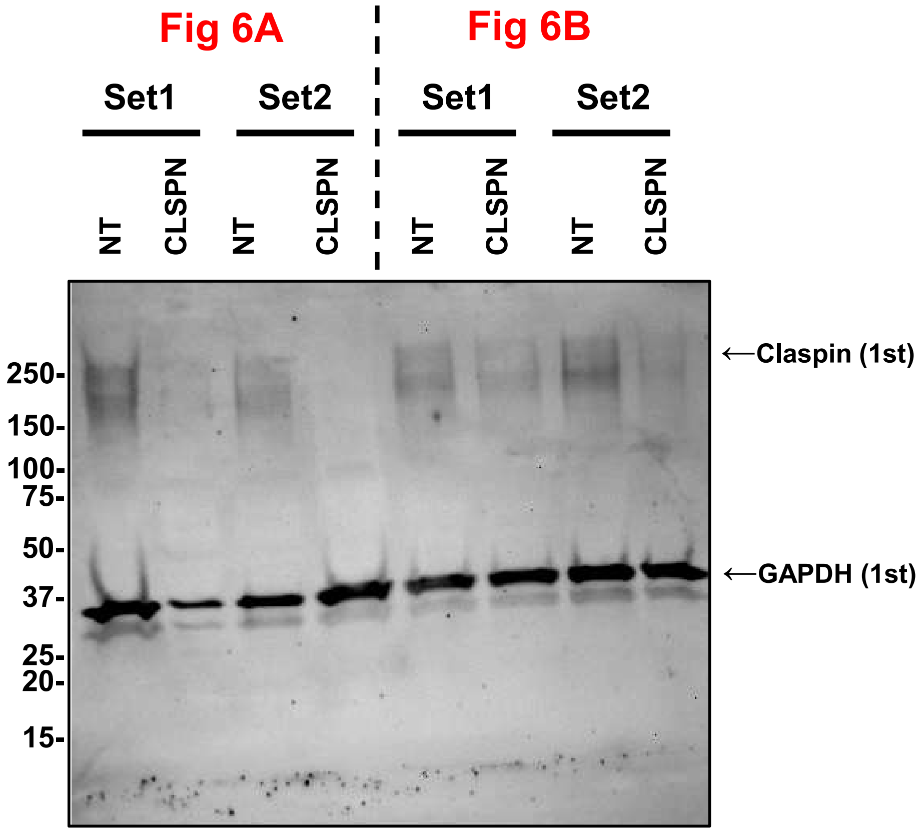

Claspin:100:7:37:0:53:35:89:25

GAPDH:100:40:54:76:57:60:60:59

Figure 6A

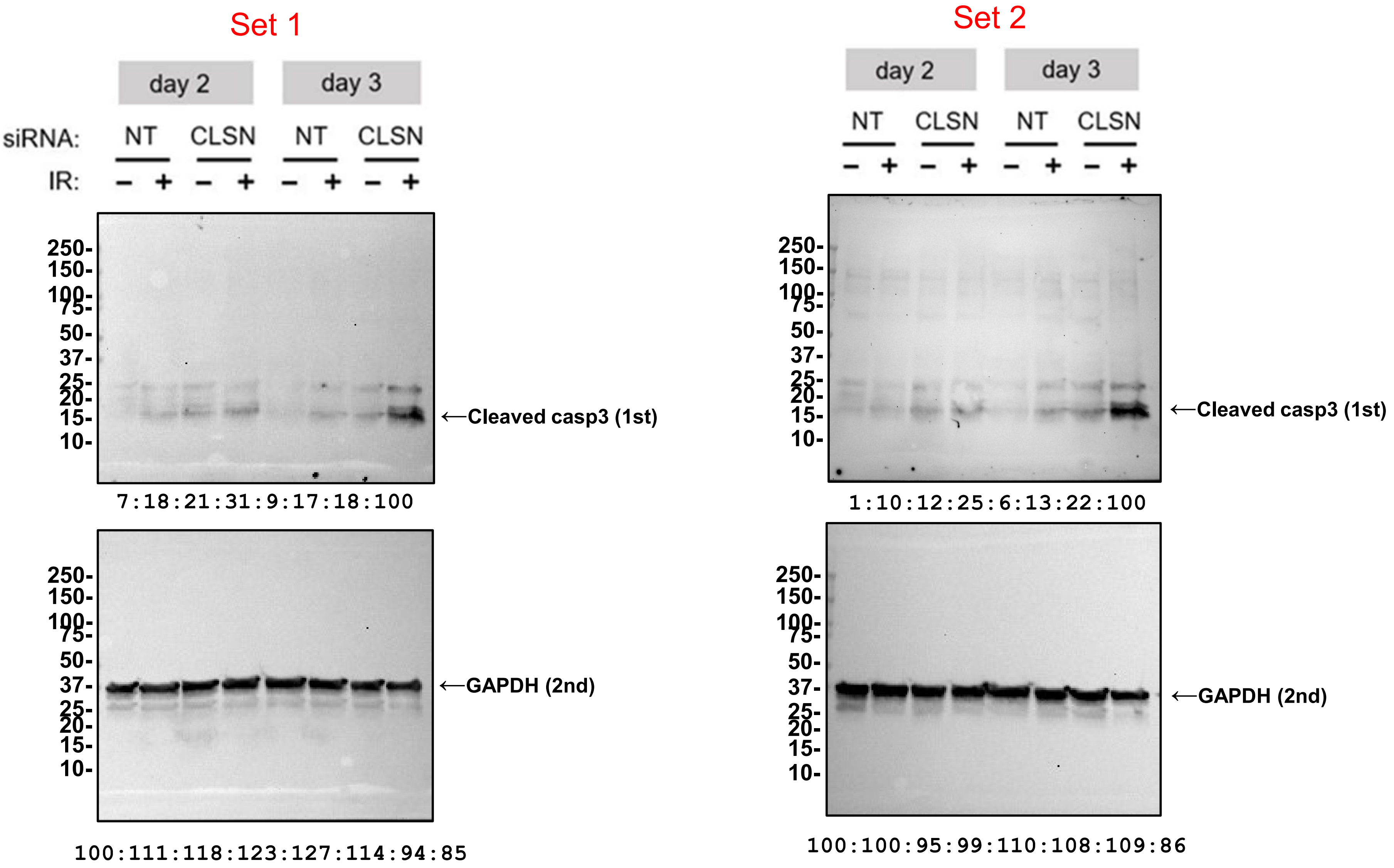

Set 1
day 2
day 3
siRNA:
NT
CLSN
NT
CLSN
IR:
– + – + – + – +
250-
150-
100-
75-
50-
37-
25-
20-
15-
10-
←Cleaved casp3 (1st)
7:18:21:31:9:17:18:100
250-
150-
100-
75-
50-
37-
25-
20-
15-
10-
←GAPDH (2nd)
100:111:118:123:127:114:94:85
Set 2
day 2
day 3
NT
CLSN
NT
CLSN
– + – + – + – +
250-
150-
100-
75-
50-
37-
25-
20-
15-
10-
←Cleaved casp3 (1st)
1:10:12:25:6:13:22:100
250-
150-
100-
75-
50-
37-
25-
20-
15-
10-
←GAPDH (2nd)
100:100:95:99:110:108:109:86

Figure 6B

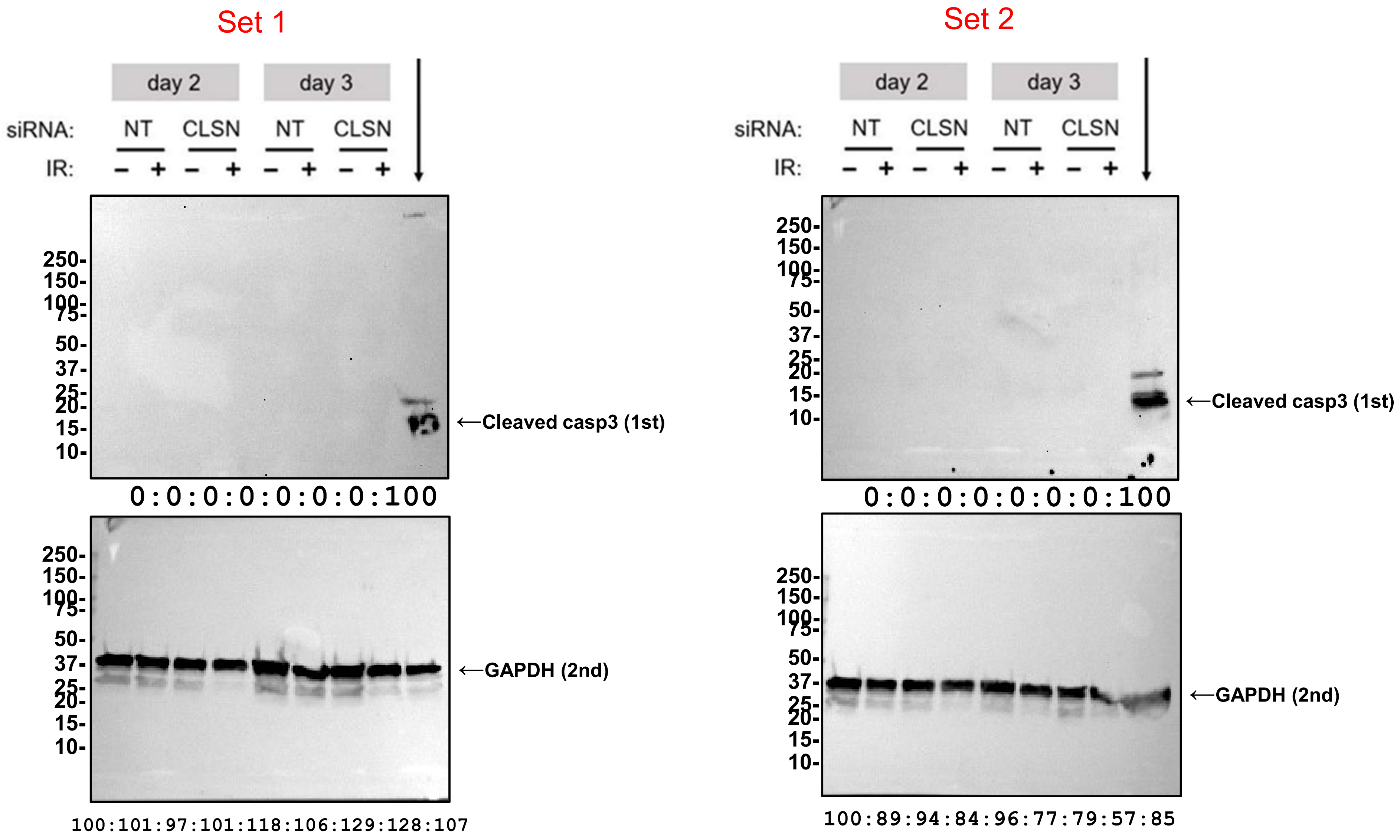

Set 1
day 2
day 3
siRNA:
NT
CLSN
NT
CLSN
IR:
– + – + – + – +
250-
150-
100-
75-
50-
37-
25-
20-
15-
10-
←Cleaved casp3 (1st)
0:0:0:0:0:0:0:0:100
←GAPDH (2nd)
100:101:97:101:118:106:129:128:107
Set 2
day 2
day 3
siRNA:
NT
CLSN
NT
CLSN
IR:
– + – + – + – +
←Cleaved casp3 (1st)
0:0:0:0:0:0:0:0:100
←GAPDH (2nd)
100:89:94:84:96:77:79:57:85

Figure S1

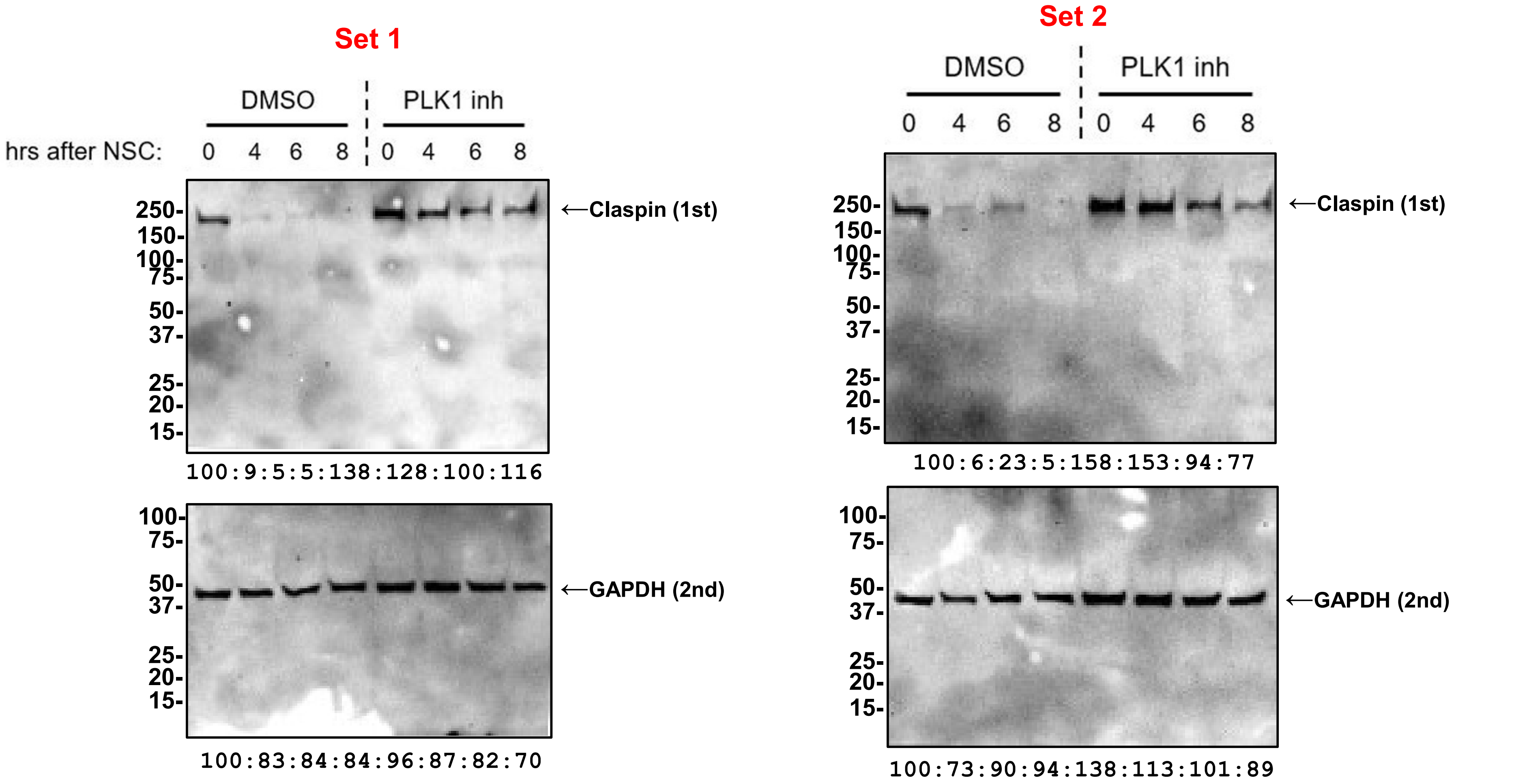

Set 1
DMSO
PLK1 inh
hrs after NSC:
0 4 6 8 0 4 6 8
250-
150-
100-
75-
50-
37-
25-
20-
15-
←Claspin (1st)
100:9:5:5:138:128:100:116
100-
75-
50-
37-
25-
20-
15-
←GAPDH (2nd)
100:83:84:84:96:87:82:70
Set 2
DMSO
PLK1 inh
0 4 6 8 0 4 6 8
250-
150-
100-
75-
50-
37-
25-
20-
15-
←Claspin (1st)
100:6:23:5:158:153:94:77
100-
75-
50-
37-
25-
20-
15-
←GAPDH (2nd)
100:73:90:94:138:113:101:89
